# Synthesis and Antimicrobial Activity of Sulfenimines Based on Pinane Hydroxythiols

**DOI:** 10.3390/antibiotics11111548

**Published:** 2022-11-04

**Authors:** Nikita O. Ilchenko, Denis V. Sudarikov, Roman V. Rumyantcev, Diana R. Baidamshina, Nargiza D. Zakarova, Monyr Nait Yahia, Airat R. Kayumov, Aleksandr V. Kutchin, Svetlana A. Rubtsova

**Affiliations:** 1 Institute of Chemistry, Federal Research Centre “Komi Scientific Centre”, Ural Branch of the Russian Academy of Sciences, Pervomayskaya St. 48, 167000 Syktyvkar, Komi Republic, Russia; 2 Institute of Fundamental Medicine and Biology, Kazan Federal University, 18 Kremlevskaya Street, 420008 Kazan, Russia; 3 G.A. Razuvaev Institute of Organometallic Chemistry of Russian Academy of Sciences, 49 Tropinina St., 603950 Nizhny Novgorod, Russia

**Keywords:** monoterpenoids, pinane, CF_3_-containing hydroxythiol, sulfenimines, antibacterial, antifungal activity

## Abstract

The widespread presence of multidrug-resistant pathogenic microorganisms challenges the development of novel chemotype antimicrobials, insensitive to microbial tools of resistance. To date, various monoterpenoids have been shown as potential antimicrobials. Among many classes of molecules with antimicrobial activity, terpenes and terpenoids are an attractive basis for the design of antimicrobials because of their low toxicity and availability for various modifications. In this work, we report on the synthesis of sulfenimines from chiral trifluoromethylated and non-fluorinated pinane-type thiols. Final compounds were obtained with yields of up to 81%. Among the 13 sulfenimines obtained, 3 compounds were able to repress the growth of both bacteria (*S. aureus*, both MSSA and MRSA; *P. aeruginosa*) and fungi (*C. albicans*) with an MIC of 8–32 µg/mL. Although compounds exhibited relatively high cytotoxicity (the therapeutic index of 3), their chemotype can be used as a starter point for the development of disinfectants and antiseptics for targeting multidrug-resistant pathogens.

## 1. Introduction

The widespread presence of multidrug-resistant bacteria and fungi challenges the development of antimicrobials of a novel chemotype, as they are insensitive to microbial tools of resistance. The acquisition of genes encoding efflux systems and enzymes that hydrolyze antimicrobials and increase biofilm formation, as well as changes in target molecules and cell wall structure, reduces the effectiveness of conventional antibiotics [1].

Among various classes of molecules able to repress the growth of pathogenic bacteria and fungi, monoterpene derivatives have a wide spectrum of antimicrobial activity [2,3,4]. Thus, the repression of the growth of both various bacteria and fungi has been reported [5,6,7,8,9,10]. The combination of terpenes with conventional antimicrobials increases the activity of the latter [11,12]. Furthermore, the fusion in one molecule of a biologically active terpene fragment and sulfur-containing functional groups, which are part of many substances with bactericidal and fungicidal activity, leads to an increase in the efficiency of the resulting thioterpenoids [2,5,13,14]. The mechanism of these synergistic effects can be a consequence of targeting the membrane itself or membrane-related proteins with terpenes. Thus, the binding site for cyclic hydrocarbons, including terpene ones [15], has been reported to be located in the cell membrane of pathogenic microorganisms. The limonene, α- and β-pinenes, and γ-terpinene are able to inhibit respiration and other energy-dependent processes localized in cell membranes of some fungi and bacteria [3,16,17,18,19]. Additionally, some derivatives of terpenes were shown to interact with membranes of eukaryotic cells [20,21].

Carane sulfenimines, sulfinimines, and *N*-substituted fluorine-containing sulfinamides, as well as pinane thiosulfonates obtained on the basis of monoterpene thiols, showed selective antimicrobial activity against yeasts *Candida albicans* and *Cryptococcus neoformans*, as well as the bacteria *Staphylococcus aureus* and *Acinetobacter baumannii* [22,23]. The introduction of a sulfenimine fragment in the structure of cephalosporin sulfoxides enhanced their inhibitory activity against cephalosporinase C. The activity was significantly affected by substituents in the sulfenimine moiety [24]. Substituted salicylic and nitrobenzylidene imines have been shown to be subjected as new chemotypes of antimicrobial drug candidates [25,26,27,28,29].

Nowadays, a third of the newly synthesized antimicrobials carry fluorine atoms [30], since the introduction of fluorine-containing groups enhances the membrane permeability and increases the resistance to biodegradation relative to their nonfluorinated analogs [31]. These modifications can lead to significant changes in interaction mechanisms of target with the drug and consequent shifts in biological activity of the latter, compared to hydrocarbon analogs [32,33]. Previously, CF_3_-containing pinane-type monoterpene hydroxythiols for further functionalization were synthesized [34].

In this work, based on 10-hydroxyisopinocampheyl thiol **1** [35], of which its CF_3_-containing analogs (10*S*)-**2** and (10*R*)-**3** were obtained earlier by our group [34], and nitrobenzaldehydes or substituted salicylic aldehydes, a series of sulfenimines were synthesized, and their antibacterial and antifungal activities, cytotoxicity, and mutagenicity were evaluated.

## 2. Results and Discussion

### 2.1. Synthesis of Sulfenimines from Pinane Hydroxythiols

**Scheme 1:**
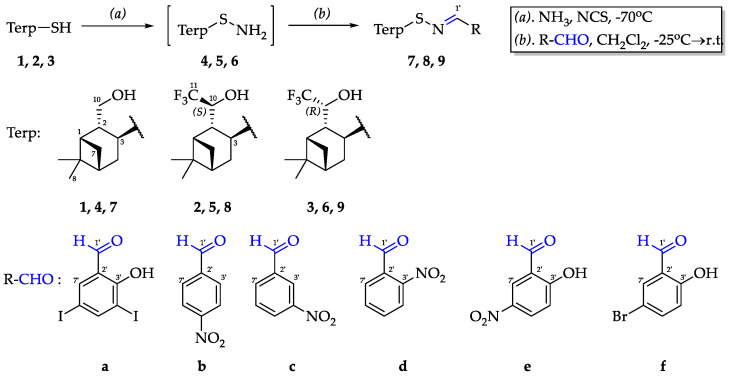
General scheme for the synthesis of sulfenimines from pinane hydroxythiols.

Thiols **1**–**3** were treated with *N*-chlorosuccinimide (NCS) in liquid ammonia according to a known procedure [23,36,37,38] to form unstable sulfenamides **4**–**6**, which entered into a condensation reaction in situ with 3,5-diiodosalicylic aldehyde (**a**), 4-nitrobenzaldehyde (**b**), 3-nitrobenzaldehyde (**c**), 2-nitrobenzaldehyde (**d**), 5-nitrosalicylic aldehyde (**e**) and 5-bromosalicylic aldehyde (**f**) to provide the corresponding sulfenimines **7a**–**f**, **8a**–**f** and **9a** in 16–81% yields without isolation of sulfenamide intermediates (Scheme 1). Since the CF_3_-containing thiol (10*R*)-**3** is synthetically less accessible than the thiol (10*S*)-**2** [34], only sulfenimine **9a** with the 3,5-diiodosalicylic moiety was synthesized on its basis.

The structures and composition of sulfenimines have been proven by NMR and IR spectroscopy, and by elemental and X-ray diffraction analysis. The IR spectra of sulfenimines **7a**–**f**, **8a**–**f** and **9a** contain absorption bands characteristic of the C=N bond in the region of 1614–1574 cm^−1^. The ^1^H and ^13^C NMR spectra contain signals from both terpene and aromatic fragments. In the ^1^H NMR spectra of **7a**–**f**, **8a**–**f** and **9a**, in comparison with the starting thiols **1**–**3**, the proton signals of the SH groups disappear, whereas proton signals of the C^1^′H=N group can be observed in the region of 8.34–8.95 ppm. In the ^13^C NMR spectra, there are signals characteristic of the C^1^′=N group in the downfield region (152.0–159.9 ppm). The ^13^C NMR spectra of CF_3_-containing sulfenimines **8a**–**f** and **9a** contain quartets of the C^11^F_3_ group in the range of 124.9–125.8 ppm (*J*_F_ = 283.0–284.2 Hz), as well as quartets of the C^10^ atom in the region of 71.1–72.6 ppm (*J*_F_ = 28.8–29.2 Hz); in contrast, in sulfenimines **7a**–**f**, the signals of the C^10^ atom are in the region of 65.9–66.4 ppm.

**Figure 1:**
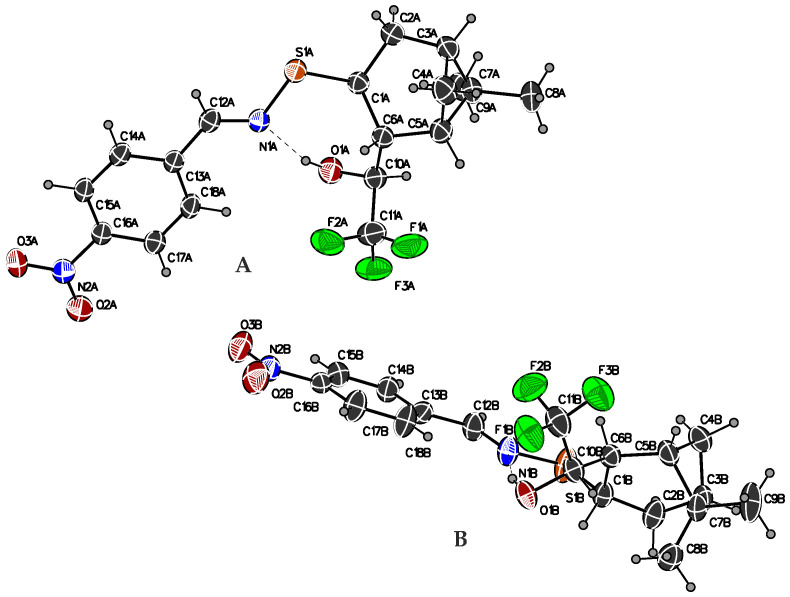
Molecular structure of two independent molecules (**A**,**B**) of compound **8b** with thermal ellipsoids drawn at the 30% probability level.

The structure and configuration of single-crystal **8b** were confirmed by X-ray diffraction analysis (Figure 1). This compound crystallizes in the chiral space group P2_1_ of the monoclinic system. There are two independent molecules (A and B) of **8b** in the asymmetric unit cell. They have the same molecular structure. The root-mean-square deviation of the nonhydrogen atomic positions of the A and B molecules is 0.213 Å. The greatest difference is the slight rotation of the SNCHR group. The dihedral angle between the corresponding planes when the molecules are superimposed is 11.28°. All atoms of the NO_2_ group, as well as the S(1), N(1) and C(12) atoms, lie in the plane of the C(13)–C(18) phenyl ring. The N-O distances in the NO_2_ group are largely aligned. This is typical for NO_2_ groups in similar compounds [23,39]. In general, the main geometric characteristics for compound **8b** are in good agreement with previously published related compounds [23,39].

Compound **8b** has an intramolecular hydrogen bond O(1)-H(1)…N(1). The data set does not allow us to refine the position of the hydrogen atom without constraints. However, the distance between the oxygen and nitrogen atoms (2.830(5) and 2.852(5) Å in molecules A and B, respectively) indicates the implementation of the H...N interaction [40].

The resulting sulfenimines **7a**–**f**, **8a**–**f** and **9a** were further subjected to an antibacterial and antifungal activity test, and an assessment of mutagenicity and cytotoxicity.

### 2.2. Antimicrobial Activity

The activity of terpenes has been shown to vary against microorganisms with various cell wall structures [2,3,4,15,16,20]. Therefore, the antimicrobial activity of the newly synthesized sulfenimine derivatives was assessed against Gram-positive bacteria (*Staphylococcus aureus* ATCC 29213 (MSSA) and a clinical isolate of *Staphylococcus aureus* resistant to methicillin (MRSA)), Gram-negative bacterium *P. aeruginosa* ATCC 27853, and a fluconazole-sensitive clinical isolate of *Candida albicans* 703. These microorganisms cause diseases of the skin, various mucosa and the respiratory tract and are characterized by a high frequency of occurrence of resistant isolates.

As could be seen from Table 1, compounds **7a, 8a**, **7c** and **8e** repressed the growth of all test microorganisms, although the activity was moderate and MIC values were generally significantly higher than those of reference antimicrobials. Notably, trifluoromethylated sulfenimines with salicylic moiety **9a** and **8f** were active only against methicillin-resistant *S. aureus* and *C. albicans*; while **9a** was even superior in activity to fluconazole.

Furthermore, **7d**, **7f** and, partially, **7b** were active only on *S. aureus* and *C. albicans*; **7d** and **7f** in the Ames test showed mutagenicity on the *Salmonella typhimurium* TA102 strain, causing point mutations and reversions [41]. Sulfenimines containing the CF_3_ group did not show mutagenicity in this test. Compound **7e** was active only on bacteria. In general, a decrease in antibacterial properties upon the introduction of a CF_3_ group in the terpene fragment of sulfenimines could be observed. Moreover, sulfenimines with a CF_3_ group in the terpene moiety and a salicylaldehyde moiety, **8a**, **9a**, **8c** and **8f**, exhibit greater antifungal activity (MIC 8–32), in contrast to the nonfluorinated analogues **7a**, **7e** and **7f** (MIC ≥ 64). Compounds **8b**, **8c** and **8d** were inactive against all test strains, suggesting that the presence of nitrobenzylidene substituents abrogates their antimicrobial activity; neither cytotoxicity nor mutagenicity was tested.

In general, the synthesized sulfenimines exhibited high cytotoxicity on the embryonic bovine lung (EBL) cells: CC_50_ values were the least toxic, whereas active sulfenimines **7c** and **8e** exceeded only the corresponding MIC by 2–3-fold. This fact makes sulfenimines suitable only as antiseptics, which is similar to benzalkonium chloride due to a similar therapeutic index (CC_50_/MIC).

## 3. Conclusions

Thus, new monoterpene sulfenimines based on monoterpene pinane thiols, including those containing a CF_3_ group, have been synthesized for the first time. The described sulfenimines, generally, have moderate antibacterial and antifungal activity and high cytotoxicity in vitro, which limits their direct application. By contrast, the revealed effects of monoterpene and aromatic moieties on the antimicrobial activity of sulfenimines allows for further modeling of compounds with given selectivity for pathogenic microorganisms.

## 4. Materials and Methods

### 4.1. General Information

IR spectra were registered on a Shimadzu IR Prestige 21 infrared Fourier spectrometer in a thin layer or in KBr pellets. ^1^H NMR and ^13^C NMR spectra were recorded on a Bruker Avance 300 spectrometer (300 and 75 MHz) in CDCl_3_ using the signal of the indicated solvent as an internal standard (See Supplementary Materials). ^13^C NMR spectra were registered in the *J*-modulation mode. The complete assignment of ^1^H and ^13^C signals was performed using 2D homo- (^1^H–^1^H COSY, ^1^H–^1^H NOESY) and heteronuclear (^1^H–^13^C HSQC, ^1^H–^13^C HMBC) experiments. ^19^F NMR spectra were recorded on a Bruker Avance 300 spectrometer (282 MHz) and on a Spinsolve 60 HF Ultra spectrometer (58 MHz) in CDCl_3_ using the signal of CF_3_COOH as an external standard. For easier interpretation of the NMR spectra, the carbon atoms of structures **7a**–**f**, **8a**–**f** and **9a** were numbered, in some cases, contrary to the recommendations of IUPAC. Elemental analysis was carried out on an EA 1110 CHNS-O automatic analyzer. Melting points were determined on a Sanyo Gallenkamp MPD350BM3.5 instrument and were not corrected. Optical rotation was measured on an automated digital polarimeter, the Optical Activity PolAAr 3001 (UK). Sorbfil plates were used for thin-layer chromatography; the visualizing agent was a solution of phosphoromolybdic acid in ethanol. Alfa Aesar silica gel (0.06–0.2 mm) was used for column chromatography. The commercially available *N*-chlorosuccinimide, 98% (Alfa Aesar); 3,5-diiodosalicylaldehyde, 98+% (Alfa Aesar); 4-nitrobenzaldehyde, 99% (Alfa Aesar); 3-nitrobenzaldehyde, 99% (Alfa Aesar); 2-nitrobenzaldehyde, 98+% (Alfa Aesar); 5-nitrosalicylaldehyde, 98% (Alfa Aesar); and 5-bromosalicylaldehyde, 98% (Alfa Aesar) were used without additional purification.

The diffraction data for compound **8b** were collected on an Oxford Xcalibur Eos diffractometer (Mo-K_α_ radiation, ω-scan technique, λ = 0.71073 Å). The intensity data were integrated by the CrysAlisPro [42] program. The SCALE3 ABSPACK algorithm [42] was used to perform absorption corrections. The structure was solved by dual methods [43] and was refined on $F_{\mathrm{hkl}}^{2}$ using the SHELXTL package [44]. All nonhydrogen atoms were refined anisotropically. All H atoms, with the exception of hydrogens of the hydroxyl groups, were placed in calculated positions and were refined using a riding model (U_iso_(H) = 1.5U_eq_(C) for CH_3_ groups and U_iso_(H) = 1.2U_eq_(C) for other groups). The H(1A) and H(1B) atoms in **8b** were located on the differential Fourier map and were refined isotropically with AFIX 147. The PhNO_2_ fragment in molecule **B** was disordered over two positions. To refine the disordered atoms, the AFIX 66, SAME, SADI, FLAT and ISOR instructions were used. CCDC 2193190 contains the supplementary crystallographic data. These data can be obtained free of charge from The Cambridge Crystallographic Data Centre via https://www.ccdc.cam.ac.uk/structures (accessed on 3 November 2022).

((1*S*,2*R*,3*S*,5*R*)-3-Mercapto-6,6-dimethylbicyclo[3.1.1]heptan-2-yl)methanol (**1**) [35], (*S*)-2,2,2-trifluoro-1-((1*S*,2*R*,3*S*,5*R*)-3-mercapto-6,6-dimethylbicyclo[3.1.1]heptan-2-yl)ethan-1-ol (**2**) and (*R*)-2,2,2-trifluoro-1-((1*S*,2*R*,3*S*,5*R*)-3-mercapto-6,6-dimethylbicyclo[3.1.1]heptan-2-yl)ethan-1-ol (**3**) [34] were synthesized according to known procedures.

### 4.2. General Procedure for the Synthesis of Sulfenimines

The procedure is based on methods for the synthesis of sulfenimines [23,36,37,38].

In a U-shaped tube, while cooling to −70 °C in an acetone bath, 7–10 mL of liquid NH_3_ (dry) was condensed. While maintaining the bath temperature, NCS (143 mg, 1.069 mmol) was carefully added. The mixture was stirred for 5 min, then thiol **1** (0.823 mmol, either **2** or **3**) dissolved in 2 mL of CH_2_Cl_2_ was introduced into the tube with a syringe. The reaction mixture was stirred for one hour, gradually increasing the temperature to −30 °C until unstable sulfenamide **2** was formed. After complete conversion of the thiol (monitored by TLC), the corresponding aldehyde (2.1 mmol, **a**–**f**) was added to the reaction mixture. As ammonia evaporated, CH_2_Cl_2_ was added. After complete evaporation of ammonia, the reaction mixture was heated to room temperature. After 12 h, the reaction mixture was filtered off on a Schott filter under reduced pressure. The organic phase was treated with a NaCl solution and extracted with CH_2_Cl_2_ (3 × 20 mL). The combined organic phases were dried over Na_2_SO_4_. The solvent was then distilled off under reduced pressure. The resulting mixture was separated by silica gel column chromatography using the same eluent systems as for TLC.

(1*S*,2*R*,3*S*,5*R*)-*N*-((*E*)-2-Hydroxy-3,5-diiodobenzylidene)-2-(hydroxymethyl)-6,6-dimethylbicyclo[3.1.1]heptane-3-sulfenamide (**7a**). Yield: 55.5%; yellow-orange powder; m.p.: 64.1 °C; ${\left[\alpha \right]}_{D}^{25}$ +56.6 (*c* 1.71, CHCl_3_); R*_f_* 0.43 (petr. ether:EtOAc, 2:1). ^1^H NMR (300 MHz, CDCl_3_, δ, ppm, *J*/Hz): 1.04 (3H, s, CH_3_-8), 1.14 (1H, d, *J* = 9.8, H-7*α*), 1.27 (3H, s, CH_3_-9), 2.04–2.11 (2H, m, H-5, C-10-OH), 2.18–2.29 (3H, m, H-1, H-4*α*, H-2), 2.50 (1H, dtd, *J* = 9.5, 6.6, 2.2, H-7*β*), 2.60–2.72 (1H, m, H-4*β*), 3.64 (1H, dt, *J* = 9.5, 6.6, H-3), 3.69–3.84 (2H, m, H-10), 7.43 (1H, d, *J* = 2.0, H-7′), 7.99 (1H, d, *J* = 2.0, H-5′), 8.35 (1H, s, H-1′), 12.45 (1H, br.s, C-3′-OH). ^13^C NMR (75 MHz, CDCl_3_, δ, ppm): 23.5 (C-8), 27.5 (C-9), 32.4 (C-7), 35.5 (C-4), 38.8 (C-6), 41.7 (C-5), 42.5 (C-3), 42.7 (C-1), 49.7 (C-2), 66.0 (C-10), 80.7 (C-6′), 86.5 (C-4′), 121.7 (C-2′), 138.7 (C-7′), 147.7 (C-5′), 157.6 (C-3′), 158.8 (C-1′). IR spectrum (KBr, ν, cm^−1^): 3404 (Ar-OH), 3383 (OH), 1574 (C=N). Elemental analysis calcd. (%) for C_17_H_21_I_2_NO_2_S: C 36.64, H 3.80, N 2.51; found: C 37.03, H 4.17, N 2.64.

(1*S*,2*R*,3*S*,5*R*)-*N*-((*E*)-2-Hydroxy-3,5-diiodobenzylidene)-6,6-dimethyl-2-((*S*)-2,2,2-trifluoro-1-hydroxyethyl)bicyclo[3.1.1]heptane-3-sulfenamide (**8a**). Yield: 69%; yellow powder; m.p.: 98.5 °C; ${\left[\alpha \right]}_{D}^{25}$ +0.2 (*c* 0.85, CHCl_3_); R*_f_* 0.53 (CHCl_3_). ^1^H NMR (300 MHz, CDCl_3_, δ, ppm, *J*/Hz): 1.06 (3H, s, CH_3_-8), 1.25 (1H, d, *J* = 9.5, H-7*α*), 1.27 (3H, s, CH_3_-9), 2.05–2.11 (1H, m, H-5), 2.20–2.21 (1H, m, H-1), 2.29–2.38 (2H, m, H-2, H-4*α*), 2.40–2.50 (1H, m, H-7*β*), 2.59–2.79 (2H, m, H-4*β*, C-10-OH), 4.05–4-21 (2H, H-3, H-10), 7.44 (1H, d, *J* = 2.0, H-7′), 8.00 (1H, d, *J* = 2.0, H-5′), 8.34 (1H, s, H-1′), 12.20 (1H, br.s, C-3′-OH). ^13^C NMR (75 MHz, CDCl_3_, δ, ppm): 23.1 (C-8), 26.9 (C-9), 30.4 (C-7), 35.1 (C-4), 38.1 (C-6), 40.7 (C-5), 40.8 (C-3), 42.4 (C-1), 46.4 (C-2), 72.6 (q, *J*_F_ = 29.2, C-10), 80.7 (C-6′), 86.6 (C-4′), 121.6 (C-2′), 124.9 (q, *J*_F_ = 283.0, C-11), 138.8 (C-7′), 147.9 (C-5′), 157.4 (C-3′), 158.4 (C-1′). ^19^F NMR (282 MHz, CDCl_3_, δ, ppm): −76.20 (3F, s, CF_3_-11). IR spectrum (KBr, ν, cm^−1^): 3460 (OH), 1576 (C=N), 1273, 1151, 1123 (CF_3_). Elemental analysis calcd. (%) for C_18_H_20_F_3_I_2_NO_2_S: C 34.58, H 3.22, N 2.24; found: C 34.95, H 3.60, N 2.08.

(1*S*,2*R*,3*S*,5*R*)-*N*-((*E*)-2-Hydroxy-3,5-diiodobenzylidene)-6,6-dimethyl-2-((*R*)-2,2,2-trifluoro-1-hydroxyethyl)bicyclo[3.1.1]heptane-3-sulfenamide (**9a**). Yield: 16%; light-yellow gummy oil; ${\left[\alpha \right]}_{D}^{26}$ +30.8 (*c* 0.4, CHCl_3_); R*_f_* 0.37 (CHCl_3_). ^1^H NMR (300 MHz, CDCl_3_, δ, ppm, *J*/Hz): 1.13 (3H, s, CH_3_-8), 1.14 (1H, d, *J* = 9.5, H-7*α*), 1.30 (3H, s, CH_3_-9), 2.05–2.13 (1H, m, H-5), 2.18 (1H, br.s, C-10-OH), 2.36–2.47 (3H, m, H-1, H-2, H-4*α*), 2.56–2.74 (2H, m, H-2, H-7*β*, H-4*β*), 3.87 (1H, td, *J* = 9.2, 6.6, H-3), 4.19–4-29 (1H, m, H-10), 7.45 (1H, d, *J* = 1.5, H-7′), 8.02 (1H, d, *J* = 1.5, H-5′), 8.40 (1H, s, H-1′), 12.32 (1H, br.s, C-3′-OH). ^13^C NMR (75 MHz, CDCl_3_, δ, ppm): 24.3 (C-8), 27.9 (C-9), 33.8 (C-7), 35.6 (C-4), 38.4 (C-6), 41.7 (C-5), 42.0 (C-1), 43.4 (C-3), 46.3 (C-2), 71.2 (q, *J*_F_ = 29.9, C-10), 80.7 (C-6′), 88.5 (C-4′), 121.6 (C-2′), 125.1 (q, *J*_F_ = 283.1, C-11), 138.9 (C-7′), 148.0 (C-5′), 157.6 (C-3′), 159.4 (C-1′). ^19^F NMR (58 MHz, CDCl_3_, δ, ppm): −74.43 (3F, d, *J* = 7.4, CF_3_-11). IR spectrum (KBr, ν, cm^−1^): 3466 (OH), 1576 (C=N), 1128, 1552, 1123 (CF_3_). Elemental analysis calcd. (%) for C_18_H_20_F_3_I_2_NO_2_S: C 34.58, H 3.22, N 2.24; found: C 34.58, H 3.14, N 2.63.

(1*S*,2*R*,3*S*,5*R*)-2-(Hydroxymethyl)-6,6-dimethyl-*N*-((*E*)-4-nitrobenzylidene)bicyclo-[3.1.1]heptane-3-sulfenamide (**7b**). Yield: 81%; yellowish oil; ${\left[\alpha \right]}_{D}^{26}$ −82.0 (*c* = 0.6, CHCl_3_); R*_f_* = 0.22 (petr. ether:EtOAc, 3:1). ^1^H, ^13^C NMR and IR spectral data correspond to those given in Ref. [37].

(1*S*,2*R*,3*S*,5*R*)-6,6-Dimethyl-*N*-((*E*)-4-nitrobenzylidene)-2-((*S*)-2,2,2-trifluoro-1-hydroxyethyl)bicyclo[3.1.1]heptane-3-sulfenamide (**8b**). Yield: 75%; light-yellow crystal; m.p.: 48.8 °C; ${\left[\alpha \right]}_{D}^{25}$−1.5 (*c* 1.41, CHCl_3_); R*_f_* 0.28 (petr. ether:EtOAc, 5:1). ^1^H NMR (300 MHz, CDCl_3_, δ, ppm, *J*/Hz): 1.09 (3H, s, CH_3_-8), 1.28 (3H, s, CH_3_-9), 1.28 (1H, d, *J* = 10.3, H-7*α*), 1.60–1.68 (1H, m, H-4*α*), 2.05–2.11 (1H, m, H-5), 2.28–2.35 (1H, m, H-1), 2.37–2.46 (1H, m, H-7β), 2.49–2.59 (1H, m, H-4β), 2.94 (1H, dt, *J* = 10.3, 3.3, H-2), 3.94–4.07 (1H, m, H-10), 4.37 (1H, dt, *J* = 10.8, 4.5, H-3), 5.58 (1H, br.d, *J* = 8.8, C-10-OH), 7.72 (2H, d, *J =* 8.8, H-3′, H-7′), 8.29 (2H, d, *J* = 8.8, H-4′, H-6′), 8.54 (1H, s, H-1′). ^13^C NMR (75 MHz, CDCl_3_, δ, ppm): 23.0 (C-8), 26.9 (C-9), 29.7 (C-7), 29.9 (C-4), 38.1 (C-6), 38.7 (C-3), 40.1 (C-5), 43.3 (C-1), 51.5 (C-2), 71.1 (q, *J*_F_ = 28.8, C-10), 124.4 (C-4′, C-6′), 125.8 (q, *J*_F_ = 283.1, C-11), 127.8 (C-3′, C-7′), 140.1 (C-2′), 148.7 (C-5′), 155.3 (C-1′). ^19^F NMR (282 MHz, CDCl_3_, δ, ppm): −76.37 (3F, s, CF_3_-11). IR spectrum (KBr, ν, cm^−1^): 3238 (OH), 1601 (C=N), 1522, 1344 (NO_2_), 1267, 1169, 1125 (CF_3_). Elemental analysis calcd. (%) for C_18_H_21_F_3_N_2_O_3_S: C 53.72, H 5.26, N 6.96; found: C 53.33, H 5.21, N 7.04. A single crystal of **8b** was grown from the hexane-Et_2_O system. A colorless prismatic crystal of the monoclinic system had a size of 0.77×0.39×0.13 mm, space group *P*2_1_, *a* = 16.6479(3), *b* = 7.08470(10), *c* = 17.9640(4) Å, *β* = *111.720(2)*°, *V* = 1968.34(7) Å^3^, Z = 4, μ = 0.212 mm^−1^, *d*_calc_ = 1.358 g/cm^3^ and F(000) = 840. A dataset of 42,548 reflections was collected at scattering angles of 2.101° < θ < 25.027°, of which 6946 were independent (R_int_ = 0.0357), including 5644 reflections with *I* > 2σ(*I*). The final refinement parameters were R_1_ = 0.0548, wR_2_ = 0.1118 (all data), R_1_ = 0.0406 and wR_2_ = 0.1006 [*I* > 2σ(*I*)], with GooF = 0.945. Δρ*_e_* = 0.266/−0.171 *e* Å^–3^; Flack parameter = −0.03(2).

(1*S*,2*R*,3*S*,5*R*)-2-(Hydroxymethyl)-6,6-dimethyl-*N*-((*E*)-3-nitrobenzylidene)bicyclo-[3.1.1]heptane-3-sulfenamide (**7c**). Yield: 53%; light-yellow oil; ${\left[\alpha \right]}_{D}^{25}$ −40.4 (*c* 2.18, CHCl_3_); R*_f_* 0.30 (PhH:EtOAc, 10:1). ^1^H NMR (300 MHz, CDCl_3_, δ, ppm, *J*/Hz): 1.04 (3H, s, CH_3_-8), 1.26 (3H, s, CH_3_-9), 1.27 (1H, d, *J* = 10.3, H-7*α*), 1.89 (1H, ddd, *J* = 14.1, 5.7, 2.9, H-4*α*), 2.02–2.15 (1H, m, H-5, H-1), 2.39–2.60 (1H, m, H-7β, H-4β), 2.67 (1H, dtd, *J* = 8.7, 5.9, 2.2, H-2), 3.37 (1H, br.s, C-10-OH), 3.64 (1H, dd, *J* = 10.3, 5.9, H-10α), 3.81–3.87 (2H, m, H-3, H-10), 7.58 (1H, t, *J =* 8.1, H-6′), 7.96 (1H, d, *J* = 8.1, H-7′), 8.22 (1H, d, *J* = 8.1, H-5′), 8.38 (1H, s, H-3′), 8.54 (1H, s, H-1′). ^13^C NMR (75 MHz, CDCl_3_, δ, ppm): 23.5 (C-8), 27.3 (C-9), 31.5 (C-7), 32.4 (C-4), 38.6 (C-6), 41.2 (C-5), 42.8 (C-3), 44.1 (C-1), 53.1 (C-2), 66.4 (C-10), 122.1 (C-3′), 124.4 (C-5′), 129.9 (C-6′), 131.9 (C-7′), 137.4 (C-2′), 148.7 (C-4′), 154.0 (C-1′). IR spectrum (KBr, ν, cm^−1^): 3402 (OH), 1614 (C=N), 1530, 1350 (NO_2_). Elemental analysis calcd. (%) for C_17_H_22_N_2_O_3_S: C 61.05, H 6.63, N 8.38; found: C 61.38, H 6.37, N 7.91.

(1*S*,2*R*,3*S*,5*R*)-6,6-Dimethyl-*N*-((*E*)-3-nitrobenzylidene)-2-((*S*)-2,2,2-trifluoro-1-hydroxyethyl)bicyclo[3.1.1]heptane-3-sulfenamide (**8c**). Yield: 24%; white powder; m.p.: 122.7 °C; ${\left[\alpha \right]}_{D}^{27}$−83.3 (*c* 0.6, CHCl_3_); R*_f_* 0.34 (petr. ether:EtOAc, 5:1). ^1^H NMR (300 MHz, CDCl_3_, δ, ppm, *J*/Hz): 1.09 (3H, s, CH_3_-8), 1.28 (3H, s, CH_3_-9), 1.28 (1H, d, *J* = 10.3, H-7*α*), 1.64 (1H, dt, *J* = 14.3, 4.0, H-4*α*), 2.05–2.11 (1H, m, H-5), 2.28–2.35 (1H, m, H-1), 2.37–2.46 (1H, m, H-7β), 2.54 (1H, ddt, *J* = 13.9, 11.3, 2.5, H-4β), 2.96 (1H, dt, *J* = 10.3, 3.3, H-2), 3.94–4.07 (1H, m, H-10), 4.37 (1H, dt, *J* = 10.8, 4.5, H-3), 5.64 (1H, br.d, *J* = 8.1, C-10-OH), 7.63 (1H, t, *J =* 7.6, H-6′), 7.97 (1H, d, *J* = 7.6, H-7′), 8.27 (1H, d, *J* = 7.6, H-5′), 8.35 (1H, s, H-3′), 8.53 (1H, s, H-1′). ^13^C NMR (75 MHz, CDCl_3_, δ, ppm): 23.0 (C-8), 26.9 (C-9), 29.7 (C-7), 29.9 (C-4), 38.1 (C-6), 38.5 (C-3), 40.1 (C-5), 43.2 (C-1), 51.4 (C-2), 71.1 (q, *J*_F_ = 28.8, C-10), 122.8 (C-3′), 125.1 (C-5′), 125.7 (q, *J*_F_ = 283.1, C-11), 130.3 (C-6′), 131.6 (C-7′), 136.6 (C-2′), 148.7 (C-4′), 155.2 (C-1′). ^19^F NMR (282 MHz, CDCl_3_, δ, ppm): −76.38 (3F, d, *J* = 7.0, CF_3_-11). IR spectrum (KBr, ν, cm^−1^): 3379 (Ar-OH), 3264 (OH), 1595 (C=N), 1530, 1350 (NO_2_), 1271, 1169, 1121 (CF_3_). Elemental analysis calcd. (%) for C_18_H_21_F_3_N_2_O_3_S: C 53.72, H 5.26, N 6.96; found: C 53.95, H 5.28, N 7.01.

(1*S*,2*R*,3*S*,5R)-2-(Hydroxymethyl)-6,6-dimethyl-*N*-((*E*)-2-nitrobenzylidene)bicyclo-[3.1.1]heptane-3-sulfenamide (**7d**). Yield: 81%; yellow oil; ${\left[\alpha \right]}_{D}^{25}$ −34.5 (*c* 1.4, CHCl_3_); R*_f_* 0.23 (PhH:EtOAc, 10:1). ^1^H NMR (300 MHz, CDCl_3_, δ, ppm, *J*/Hz): 1.03 (3H, s, CH_3_-8), 1.25 (3H, s, CH_3_-9), 1.26 (1H, d, *J* = 9.8, H-7*α*), 1.86 (1H, ddd, *J* = 14.3, 5.5, 2.9, H-4*α*), 2.02–2.12 (2H, m, H-5, H-1), 2.39–2.59 (2H, m, H-7*β*, H-4*β*), 2.68 (1H, dtd, *J* = 8.7, 5.9, 2.2, H-2), 3.45 (1H, br.s, C-10-OH), 3.60 (1H, dd, *J* = 9.5, 6.6, H-10α), 3.78–3.90 (2H, m, H-10*β*, H-3), 7.52 (1H, td, *J* = 7.9, 1.7, H-5′), 7.66 (1H, t, *J* = 7.8, H-6′), 7.95 (1H, dd, *J* = 7.9, 1.7, H-7′), 8.00 (1H, d, *J* = 7.9, H-4′), 8.94 (1H, s, H-1′). ^13^C NMR (75 MHz, CDCl_3_, δ, ppm): 23.5 (C-8), 27.2 (C-9), 31.4 (C-7), 32.0 (C-4), 38.6 (C-6), 41.2 (C-5), 42.6 (C-3), 44.2 (C-1), 53.2 (C-2), 66.4 (C-10), 124.6 (C-4′), 128.4 (C-7′), 130.3 (C-5′), 130.4 (C-2′), 133.6 (C-6′), 147.7 (C-3′), 152.0 (C-1′). IR spectrum (KBr, ν, cm^−1^): 3395 (OH), 1605 (C=N). Elemental analysis calcd. (%) for C_17_H_22_N_2_O_3_S: C 61.05, H 6.63, N 8.38; found: C 61.29, H 7.04, N 8.19.

(1*S*,2*R*,3*S*,5*R*)-6,6-Dimethyl-*N*-((*E*)-2-nitrobenzylidene)-2-((*S*)-2,2,2-trifluoro-1-hydroxyethyl)bicyclo[3.1.1]heptane-3-sulfenamide (**8d**). Yield: 52%; yellow oil; ${\left[\alpha \right]}_{D}^{25}$ −58.8 (*c* 1.69, CHCl_3_); R*_f_* 0.35 (PhH). ^1^H NMR (300 MHz, CDCl_3_, δ, ppm, *J*/Hz): 1.08 (3H, s, CH_3_-8), 1.28 (3H, s, CH_3_-9), 1.32 (1H, d, *J* = 10.6, H-7*α*), 1.60–1.68 (1H, m, H-4*α*), 2.05–2.11 (1H, m, H-5), 2.29–2.36 (1H, m, H-1), 2.37–2.46 (1H, m, H-7β), 2.49–2.59 (1H, m, H-4β), 2.97 (1H, dt, *J* = 10.3, 3.3, H-2), 3.93–4.06 (1H, m, H-10), 4.34 (1H, dt, *J* = 10.3, 4.4, H-3), 5.57 (1H, br.d, *J* = 8.1, C-10-OH), 7.58 (1H, t, *J* = 7.9, H-5′), 7.72 (1H, t, *J* = 7.9, H-6′), 7.85 (1H, d, *J* = 7.9, H-7′), 8.06 (1H, d, *J* = 7.9, H-4′), 8.95 (1H, s, H-1′). ^13^C NMR (75 MHz, CDCl_3_, δ, ppm): 23.0 (C-8), 26.8 (C-9), 29.5 (C-7), 29.9 (C-4), 38.1 (C-6), 38.7 (C-3), 40.1 (C-5), 43.2 (C-1), 51.5 (C-2), 71.1 (q, *J*_F_ = 28.8, C-10), 124.8 (C-4′), 125.7 (q, *J*_F_ = 283.0, C-11), 128.3 (C-7′), 130.0 (C-2′), 130.9 (C-5′), 134.0 (C-6′), 147.7 (C-3′), 153.4 (C-1′). ^19^F NMR (282 MHz, CDCl_3_, δ, ppm): −76.12 (3F, d, *J* = 6.6, CF_3_-11). IR spectrum (KBr, ν, cm^−1^): 3364 (OH), 1605 (C=N), 1528, 1344 (NO_2_), 1269, 1169, 1123 (CF_3_). Elemental analysis calcd. (%) for C_18_H_21_F_3_N_2_O_3_S: C 53.72, H 5.26, N 6.96; found: C 53.98, H 5.31, N 6.60.

(1*S*,2*R*,3*S*,5*R*)-*N*-((*E*)-2-Hydroxy-5-nitrobenzylidene)-2-(hydroxymethyl)-6,6-dimethylbicyclo[3.1.1]heptane-3-sulfenamide (**7e**). Yield: 66%; yellow powder; m.p.: 108.5 °C; ${\left[\alpha \right]}_{D}^{25}$ +65.4 (*c* 1.17, CHCl_3_); R*_f_* 0.29 (PhH:EtOAc, 10:1). ^1^H NMR (300 MHz, CDCl_3_, δ, ppm, *J*/Hz): 1.05 (3H, s, CH_3_-8), 1.15 (1H, d, *J* = 9.5, H-7*α*), 1.27 (3H, s, CH_3_-9), 2.04–2.11 (2H, m, H-5, C-10-OH), 2.18–2.35 (3H, m, H-1, H-4*α*, H-2), 2.46 (1H, dtd, *J* = 9.5, 6.6, 2.2 H-7*β*), 2.58–2.68 (1H, m, H-4*β*), 4.36 (1H, dt, *J* = 10.3, 6.6, H-3), 3.73–3.86 (2H, m, H-10), 7.01 (1H, d, *J* = 8.8, H-4′), 8.13–8.18 (2H, m, H-5′, H-7′), 8.60 (1H, s, H-1′), 12.40 (1H, br.s, C-3′-OH). ^13^C NMR (75 MHz, CDCl_3_, δ, ppm): 23.4 (C-8), 27.4 (C-9), 32.2 (C-7), 35.0 (C-4), 38.8 (C-6), 41.6 (C-5), 42.4 (C-3), 42.8 (C-1), 50.0 (C-2), 65.9 (C-10), 117.7 (C-4′), 119.4 (C-2′), 126.2 (C-7′), 126.9 (C-5′), 140.3 (C-6′), 158.8 (C-1′), 163.9 (C-3′). IR spectrum (KBr, ν, cm^−1^): 3553 (Ar-OH), 3537 (OH), 1593 (C=N), 1520, 1339 (NO_2_). Elemental analysis calcd. (%) for C_17_H_22_N_2_O_4_S: C 58.27, H 6.33, N 7.99; found: C 57.87, H 5.85, N 7.60.

(1*S*,2*R*,3*S*,5*R*)-*N*-((*E*)-2-Hydroxy-5-nitrobenzylidene)-6,6-dimethyl-2-((*S*)-2,2,2-trifluoro-1-hydroxyethyl)bicyclo[3.1.1]heptane-3-sulfenamide (**8e**). Yield: 41%; yellow gummy oil; ${\left[\alpha \right]}_{D}^{25}$ +38.9 (*c* 1.14, CHCl_3_); R*_f_* 0.30 (petr. ether:EtOAc, 4:1). ^1^H NMR (300 MHz, CDCl_3_, δ, ppm, *J*/Hz): 1.07 (3H, s, CH_3_-8), 1.27 (3H, s, CH_3_-9), 1.30 (1H, d, *J* = 9.0, H-7*α*), 2.05–2.13 (1H, m, H-5), 2.20–2.29 (2H, m, H-1, H-4*α*), 2.40–2.51 (3H, m, H-2, H-7*β*, C-10-OH), 2.64–2.76 (1H, m, H-4*β*), 4.09–4.23 (2H, m, H-3, H-10), 7.02 (1H, d, *J* = 8.8, H-4′), 8.14–8.19 (2H, m, H-5′, H-7′), 8.59 (1H, s, H-1′), 12.10 (1H, br.s, C-3′-OH). ^13^C NMR (75 MHz, CDCl_3_, δ, ppm): 23.1 (C-8), 26.8 (C-9), 30.0 (C-7), 34.3 (C-4), 38.0 (C-6), 40.3 (C-5), 40.6 (C-3), 42.4 (C-1), 46.7 (C-2), 72.4 (q, *J*_F_ = 29.2, C-10), 117.8 (C-4′), 119.4 (C-2′), 125.0 (q, *J*_F_ = 283.1, C-11), 126.4 (C-7′), 127.0 (C-5′), 140.3 (C-6′), 158.7 (C-1′), 163.9 (C-3′). ^19^F NMR (282 MHz, CDCl_3_, δ, ppm): −76.17 (1F, d, *J* = 6.5, CF_3_-11). IR spectrum (KBr, ν, cm^−1^): 3509 (OH), 1593 (C=N), 1522, 1341 (NO_2_), 1277, 1167, 1123 (CF_3_). Elemental analysis calcd. (%) for C_18_H_21_F_3_N_2_O_4_S: C 51.67, H 5.06, N 6.70; found: C 51.69, H 5.27, N 6.82.

(1*S*,2*R*,3*S*,5*R*)-*N*-((*E*)-5-Bromo-2-hydroxybenzylidene)-2-(hydroxymethyl)-6,6-dimethylbicyclo[3.1.1]heptane-3-sulfenamide (**7f**). Yield: 61%; light-yellow gummy oil; ${\left[\alpha \right]}_{D}^{26}$ +55.9 (*c* 0.2, CHCl_3_); R*_f_* 0.36 (PhH:EtOAc, 5:1). ^1^H NMR (300 MHz, CDCl_3_, δ, ppm, *J*/Hz): 1.04 (3H, s, CH_3_-8), 1.14 (1H, d, *J* = 9.9, H-7*α*), 1.27 (3H, s, CH_3_-9), 1.63 (1H, br.s, C-10-OH), 2.03–2.09 (1H, m, H-5), 2.18–2.34 (3H, m, H-1, H-4*α*, H-2), 2.44–2.53 (1H, m, H-7*β*), 2.56–2.66 (1H, m, H-4*β*), 3.59 (1H, dt, *J* = 9.9, 6.6, H-3), 3.69–3.85 (2H, m, H-10), 6.84 (1H, d, *J* = 8.8, H-4′), 7.27 (1H, d, *J* = 2.2, H-7′), 7.35 (1H, dd, *J* = 8.8, 2.2, H-5′), 8.48 (1H, s, H-1′), 11.51 (1H, br.s, C-3′-OH). ^13^C NMR (75 MHz, CDCl_3_, δ, ppm): 23.4 (C-8), 27.5 (C-9), 32.4 (C-7), 35.1 (C-4), 38.8 (C-6), 41.7 (C-5), 42.4 (C-1), 42.8 (C-3), 50.1 (C-2), 66.1 (C-10), 110.8 (C-6′), 118.9 (C-4′), 121.5 (C-2′), 132.5 (C-7′), 134.3 (C-5′), 157.8 (C-3′), 159.7 (C-1′). IR spectrum (KBr, ν, cm^−1^): 3341 (OH), 1587 (C=N). Elemental analysis calcd. (%) for C_17_H_22_BrNO_2_S: C 53.13, H 5.77, N 3.64; found: C 53.10, H 5.71, N 3.55.

(1*S*,2*R*,3*S*,5*R*)-*N*-((*E*)-2-Hydroxy-5-bromobenzylidene)-6,6-dimethyl-2-((*S*)-2,2,2-trifluoro-1-hydroxyethyl)bicyclo[3.1.1]heptane-3-sulfenamide (**8f**). Yield: 38%; yellow oil; m.p.: 122.7 °C; ${\left[\alpha \right]}_{D}^{26}$ +29.0 (*c* 1.3, CHCl_3_); R*_f_* 0.34 (PhH:CH_2_Cl_2_, 2:1). ^1^H NMR (300 MHz, CDCl_3_, δ, ppm, *J*/Hz): 1.05 (3H, s, CH_3_-8), 1.25 (1H, d, *J* = 9.9, H-7*α*), 1.25 (3H, s, CH_3_-9), 2.02–2.09 (1H, m, H-5), 2.18–2.34 (2H, m, H-1, H-4*α*), 2.37–2.50 (2H, m, H-7*β*, H-2), 2.55–2.80 (2H, m, H-4*β*, C-10-OH), 4.00–4.20 (2H, m, H-3, H-10), 6.83 (1H, d, *J* = 8.8, H-4′), 7.27 (1H, s, H-7′), 7.34 (1H, d, *J* = 8.8, 2.2, H-5′), 8.45 (1H, s, H-1′), 11.25 (1H, br.s, C-3′-OH). ^13^C NMR (75 MHz, CDCl_3_, δ, ppm): 23.0 (C-8), 26.9 (C-9), 30.3 (C-7), 34.7 (C-4), 38.1 (C-6), 40.5 (C-3), 40.8 (C-5), 42.5 (C-1), 46.6 (C-2), 72.6 (q, *J*_F_ = 29.8, C-10), 110.9 (C-6′), 118.9 (C-4′), 121.4 (C-2′), 125.0 (q, *J*_F_ = 284.2, C-11), 132.7 (C-7′), 134.5 (C-5′), 157.7 (C-3′), 159.9 (C-1′). ^19^F NMR (58 MHz, CDCl_3_, δ, ppm): −74.11 (3F, d, *J* = 6.1, CF_3_-11). IR spectrum (KBr, ν, cm^−1^): 3455 (OH), 1589 (C=N), 1271, 1171, 1123 (CF_3_), 1076 (CBr). Elemental analysis calcd. (%) for C_18_H_21_BrF_3_NO_2_S: C 47.80, H 4.68, N 3.10; found: C 47.53, H 4.31, N 3.07.

### 4.3. Antibacterial Activity

Minimum inhibitory concentrations (MICs) of compounds were determined by the broth microdilution assay in 96-well plates (Eppendorf, Hamburg, Germany) according to the EUCAST rules for antimicrobial susceptibility testing [45] in full Mueller–Hinton broth (MH). Briefly, the bacterial suspension containing 10^8^ CFUs/mL was subsequently diluted to 1:300 with MH broth in microwell plates to obtain a 10^6^ cells/mL suspension, and then incubated at 37 °C for 24 h. The stock solutions of compounds to be tested were prepared in DMSO and added to the final concentrations of compounds to be tested, which ranged from 1 to 1048 µg/mL. The MIC was determined as the lowest concentration of an antibiotic for which no visible bacterial growth could be observed after 24 h of incubation. The assessment was performed five times and the typical (median) value was considered as the MIC.

### 4.4. Antifungal Activity

MICs on *C. albicans* were determined using the broth microdilution method in 96-well plates (Eppendorf) with MH broth, as recommended in the protocol CLSI M27-A3 [46]. *C. albicans* was grown overnight and diluted with MH broth until a density of 10^7^ cells/mL was reached, obtaining the working solution. Then, 2-fold serial dilutions of compounds in concentrations from 1 to 1024 μg/mL were prepared in MH broth and seeded with fungi (1% *v*/*v* of working solution) with subsequent incubation at 37 °C for 24 h. The MIC was defined as the lowest concentration of the compound at which no visible growth could be seen. The assessment was performed five times and the typical (median) value was considered as the MIC.

### 4.5. Mutagenicity and Cytotoxicity

The mutagenicity of compounds was evaluated in the Ames test with *S. typhimurium* TA98, TA100 and TA102 strains, as described in [41]. The spot-test modification was applied to avoid false-negative results due to the antibacterial activity of compounds. The tested compound was considered to be mutagenic if the number of revertant colonies increased more than 2 times when close to the filter paper with the compound.

The cytotoxicity of compounds was determined using the microtetrazolium test (MTT) on BFL cells. The cells were cultured in DMEM—Dulbecco’s Modified Eagle’s Medium (Sigma Aldrich, St. Louis, MO, USA)—that was supplemented with 10% FBS, 2 mM of *L*-glutamine, 100 µg/mL of penicillin and 100 µg/mL of streptomycin. Cells were seeded in 96-well plates with a density of 3000 cells per well and left overnight to allow for the attachment. Next, cells were cultured at 37 °C and 5% CO_2_ in the presence of compounds of interest at various concentrations from 1.25 to 160 µg/mL. After 24 h of cultivation, the cells were subjected to the MTT assay. The formazan was solubilized by DMSO and the optical density was measured on the Tecan Infinite 200Pro at 570 nm. The concentration required to inhibit cellular dehydrogenase activity by 50% (CC_50_ value) was calculated by using the GraphPad Prism 6.0 software.

## Figures and Tables

**Table 1:** Antibacterial and antifungal activities, and cytotoxicity and mutagenicity of sulfenimines.

Compound	MIC, µg/mL				IC_50_ EBL, μg/mL	Mutagenicity in the Ames Test
	*S. aureus* ATCC 29213 (MSSA)	*S. aureus* Clinical Isolate (MRSA)	*P. aeruginosa* ATCC 27853	*C. albicans* 703 Clinical Isolate		
**7a**	8	8	8	64	21 ± 2.7	NF *
**8a**	16	16	32	32	23 ± 3.8	NF
**9a**	256	8	1024	8	14 ± 3.5	NF
**7b**	64	>1024	>1024	64	20 ± 2.8	NF
**8b**	>1024	>1024	>1024	>1024	ND	ND
**7c**	32	32	32	32	45 ± 10.1	NF
**8c**	512	>1024	512	>1024	ND	ND
**7d**	32	32	>1024	512	18 ± 4.5	TA 102
**8d**	>1024	>1024	>1024	1024	ND	ND
**7e**	32	64	32	>1024	14 ± 4.6	NF
**8e**	16	32	32	16	35 ± 9.1	NF
**7f**	32	32	1024	64	9 ± 1.9	TA 102
**8f**	256	64	1024	16	14 ± 3.1	NF
Amikacin	4	4	4	ND	ND	ND
Ampicillin	0.5	>1024	>1024	ND	ND	ND
Ciprofloxacin	2	4	4	ND	ND	ND
Fluconazole	ND *	ND	ND	16	ND	ND
Benzalkonium chloride	1	1	4	0.5	1 ± 0.3	ND

* ND—not determined; NF—not found.

## Data Availability

Not applicable.

## Supplementary Materials

The following are available online at https://www.mdpi.com/article/10.3390/antibiotics11111548/s1. The ^1^H NMR, ^13^C NMR and IR spectra of novel compounds.

## References

[1] Blair J.M.A., Webber M.A., Baylay A.J., Ogbolu D.O., Piddock L.J.V. (2015). Molecular mechanisms of antibiotic resistance. Nat. Rev. Microbiol..

[2] Nikitina L.E., Artemova N.P., Startseva V.A. (2011). Natural and Thiomodified Monoterpenoids (Russian Edition).

[3] Griffin S.G., Wyllie S.G., Markham J.L., Leach D.N. (1999). The Role of Structure and Molecular Properties of Terpenoids in Determining Their Antimicrobial Activity. Flavour Fragr. J..

[4] Griffin S.G., Wyllie S.G., Markham J.L. (2005). Antimicrobially Active Terpenes Cause K+ Leakage in *E. coli* Cells. J. Essent. Oil Res..

[5] Gavrilov V.V., Startseva V., Nikitina L., Lodochnikova O.A., Gnezdilov O., Lisovskaya S., Glushko N., Klimovitskii E.N. (2010). Synthesis and antifungal activity of sulfides, sulfoxides, and sulfones based on (1S)-(-)-β-pinene. Pharm. Chem. J..

[6] Mancuso M., Catalfamo M., Laganà P., Rappazzo A.C., Raymo V., Zampino D., Zaccone R. (2019). Screening of antimicrobial activity of citrus essential oils against pathogenic bacteria and *Candida* strains. Flavour Fragr. J..

[7] Patil S.P., Kumbhar S.T. (2018). Evaluation of terpene-rich extract of Lantana camara L. leaves for antimicrobial activity against mycobacteria using Resazurin Microtiter Assay (REMA). Beni-Suef Univ. J. Basic Appl. Sci..

[8] Guimarães A.C., Meireles L.M., Lemos M.F., Guimarães M.C.C., Endringer D.C., Fronza M., Scherer R. (2019). Antibacterial Activity of Terpenes and Terpenoids Present in Essential Oils. Molecules.

[9] Kifer D., Mužinić V., Klarić M.Š. (2016). Antimicrobial Potency of Single and Combined Mupirocin and Monoterpenes, Thymol, Menthol and 1,8-Cineole against Staphylococcus Aureus Planktonic and Biofilm Growth. J. Antibiot..

[10] Cordeiro L., Figueiredo P., Souza H., Sousa A., Andrade-Júnior F., Barbosa-Filho J., Lima E. (2020). Antibacterial and Antibiofilm Activity of Myrtenol against Staphylococcus aureus. Pharmaceuticals.

[11] Selvaraj A., Valliammai A., Sivasankar C., Suba M., Sakthivel G., Pandian S.K. (2020). Antibiofilm and Antivirulence Efficacy of Myrtenol Enhances the Antibiotic Susceptibility of Acinetobacter Baumannii. Sci. Rep..

[12] Zacchino S.A., Butassi E., Cordisco E., Svetaz L.A. (2017). Hybrid combinations containing natural products and antimicrobial drugs that interfere with bacterial and fungal biofilms. Phytomedicine.

[13] Sofronov A.V., Nizamov I.S., Almetkina L.A., Nikitina L.E., Fatyhova D.G., Zelenikhin P.V., Il’Inskaya O.N., Cherkasov R.A. (2010). Monoterpenoids dithiophosphates. Synthesis and biological activity. Russ. J. Gen. Chem..

[14] Nizamov I.S., Al’metkina L.A., Gabdullina G.T., Shamilov R.R., Sofronov A.V., Nikitina L.E., Lisovskaya S.A., Glushko N.I., Cherkasov R.A. (2012). Chiral Phosphorus Dithio Acids Derived from (1S,2S,3S,5R)-(+)-Isopinocampheol. Synthesis and Fungicidal Activity. Russ. Chem. Bull..

[15] Sikkema J., de Bont J.A., Poolman B. (1995). Mechanisms of Membrane Toxicity of Hydrocarbons. Microbiol. Rev..

[16] Uribe S., Pena A. (1990). Toxicity of allelopathic monoterpene suspensions on yeast dependence on droplet size. J. Chem. Ecol..

[17] Šturm L., Poklar Ulrih N. (2020). Propolis Flavonoids and Terpenes, and Their Interactions with Model Lipid Membranes: A Review. Advances in Biomembranes and Lipid Self-Assembly.

[18] Nogueira J.O.E., Campolina G.A., Batista L.R., Alves E., Caetano A.R.S., Brandão R.M., Nelson D.L., Cardoso M.D.G. (2021). Mechanism of Action of Various Terpenes and Phenylpropanoids against *Escherichia coli* and *Staphylococcus aureus*. FEMS Microbiol. Lett..

[19] Yang S.-K., Yusoff K., Ajat M., Yap W.-S., Lim S.-H.E., Lai K.-S. (2021). Antimicrobial Activity and Mode of Action of Terpene Linalyl Anthranilate against Carbapenemase-Producing Klebsiella Pneumoniae. J. Pharm. Anal..

[20] Nikitina L.E., Artemova N.P., Startseva V.A., Fedyunina I.V., Klochkov V.V. (2017). Biological Activity of S-Containing Monoterpenoids. Chem. Nat. Compd..

[21] Mendanha S., Moura S.S., Anjos J.L., Valadares M.C., Alonso A. (2013). Toxicity of terpenes on fibroblast cells compared to their hemolytic potential and increase in erythrocyte membrane fluidity. Toxicol. In Vitro.

[22] Grebyonkina O.N., Lezina O.M., Izmest’Ev E.S., Sudarikov D.V., Pestova S.V., Rubtsova S.A., Kutchin A.V. (2017). Synthesis of new monoterpene sulfonic acids and their derivatives. Russ. J. Org. Chem..

[23] Sudarikov D.V., Krymskaya Y.V., Il’chenko N.O., Slepukhin P.A., Rubtsova S.A., Kutchin A.V. (2018). Synthesis and Biological Activity of Fluorine-Containing Amino Derivatives Based on 4-Caranethiol. Russ. Chem. Bull..

[24] Zhang K., Ding H.-W., Ju H., Huang Q., Zhang L.-J., Song H.-R., Fu D.-C. (2015). Design, Synthesis and Biological Evaluation of Sulfenimine Cephalosporin Sulfoxides as β-Lactamase Inhibitors. Chin. Chem. Lett..

[25] Xu S.-P., Lv P.-C., Shi L., Zhu H.-L. (2010). Design, Synthesis, and Pharmacological Investigation of Iodined Salicylimines, New Prototypes of Antimicrobial Drug Candidates. Arch. Pharm. Chem. Life Sci..

[26] Ceramella J., Iacopetta D., Catalano A., Cirillo F., Lappano R., Sinicropi M.S. (2022). A Review on the Antimicrobial Activity of Schiff Bases: Data Collection and Recent Studies. Antibiotics.

[27] Qiu Y., Chan S.T., Lin L., Shek T.L., Tsang T.F., Barua N., Zhang Y., Ip M., Chan P.K.-S., Blanchard N. (2019). Design, synthesis and biological evaluation of antimicrobial diarylimine and –amine compounds targeting the interaction between the bacterial NusB and NusE proteins. Eur. J. Med. Chem..

[28] da Silva C.M., da Silva D.L., Modolo L., Alves R.B., de Resende M.A., Martins C.V., de Fátima Â. (2011). Schiff bases: A short review of their antimicrobial activities. J. Adv. Res..

[29] Sayyed M., Mokle S., Bokhare M., Mankar A., Surwase S., Bhusare S., Vibhute Y. (2006). Synthesis of Some New 2,3-Diaryl-1,3-Thiazolidin-4-Ones as Antibacterial Agents. Arkivoc.

[30] Johnson B.M., Shu Y.-Z., Zhuo X., Meanwell N.A. (2020). Metabolic and Pharmaceutical Aspects of Fluorinated Compounds. J. Med. Chem..

[31] Meanwell N.A. (2011). Synopsis of Some Recent Tactical Application of Bioisosteres in Drug Design. J. Med. Chem..

[32] Isanbor C., O’Hagan D. (2006). Fluorine in Medicinal Chemistry: A Review of Anti-Cancer Agents. J. Fluor. Chem..

[33] Hagmann W.K. (2008). The Many Roles for Fluorine in Medicinal Chemistry. J. Med. Chem..

[34] Ilchenko N.O., Sudarikov D.V., Slepukhin P.A., Rubtsova S.A., Kutchin A.V. (2021). Synthesis of Chiral CF_3_-Contaning Pinane-Type Hydroxythiols. ChemistrySelect.

[35] Martínez-Ramos F., Vargas-Díaz M.E., Chacón-García L., Tamariz J., Joseph-Nathan P., Zepeda L.G. (2001). Highly Diastereoselective Nucleophilic Additions Using a Novel Myrtenal-Derived Oxathiane as a Chiral Auxiliary. Tetrahedron Asymmetry.

[36] Sudarikov D.V., Krymskaya Y.V., Melekhin A.K., Shevchenko O.G., Rubtsova S.A. (2021). Synthesis and Antioxidant Activity of Monoterpene Nitrobenzylidenesulfenimines. Chem. Pap..

[37] Sudarikov D.V., Krymskaya Y.V., Shevchenko O.G., Slepukhin P.A., Rubtsova S.A., Kutchin A.V. (2019). Synthesis and Antioxidant Activity of Carane and Pinane Based Sulfenimines and Sulfinimines. Chem. Biodivers..

[38] Yang T.-K., Chen R.-Y., Lee D.-S., Peng W.-S., Jiang Y.-Z., Mi A.-Q., Jong T.-T. (1994). Application of New Camphor-Derived Mercapto Chiral Auxiliaries to the Synthesis of Optically Active Primary Amines. J. Org. Chem..

[39] Mloston G., Romanski J., Linden A., Heimgartner H. (1995). Erstes Beispiel einer H-Verschiebung in ‘Thiocarbonyl-aminiden’ (N-(Alkylidensulfonio)aminiden). Helv. Chim. Acta.

[40] Andrade L.A.F., Silla J.M., Freitas M.P. (2014). The Gauche Effect Is Governed by Internal Hydrogen Bond in 2-Amino-2-Methyl-Propanol. J. Mol. Struct..

[41] McCann J., Ames B.N. (1976). A Simple Method for Detecting Environmental Carcinogens as Mutagens. Ann. N. Y. Acad. Sci..

[42] SAINT (2014). Data Reduction and Correction Program.

[43] Sheldrick G.M. (2015). *SHELXT*—Integrated space-group and crystal-structure determination. Acta Crystallogr. Sect. A Found. Adv..

[44] Sheldrick G.M. (2015). Crystal structure refinement with *SHELXL*. Acta Crystallogr. Sect. C Struct. Chem..

[45] Leclercq R., Cantón R., Brown D.F.J., Giske C.G., Heisig P., MacGowan A.P., Mouton J.W., Nordmann P., Rodloff A.C., Rossolini G.M. (2013). EUCAST expert rules in antimicrobial susceptibility testing. Clin. Microbiol. Infect..

[46] Rex J.H., Clinical and Laboratory Standards Institute, Clinical and Laboratory Standards Institute (2008). Reference Method for Broth Dilution Antifungal Susceptibility Testing of Yeats: Approved Standard.

