# Supplementary material for: Synthesis and Antimicrobial Activity of Sulfenimines Based on Pinane Hydroxythiols
Source: Antibiotics (Basel). 2022 Nov 4;11(11):1548. doi: 10.3390/antibiotics11111548 (PMC9686613; doi:10.3390/antibiotics11111548)

Antibiotics

Supplementary Materials

Synthesis and antimicrobial activity of sulfenimines based on pinane hydroxythiols

Nikita O. Ilchenko, Denis V. Sudarikov, Roman V. Rumyantcev, Diana R. Baidamshina, Nargiza D. Zakarova, Monyr Nait Yahia, Airat R. Kayumov, Aleksandr V. Kutchin and Svetlana A. Rubtsova

^{1}H, ^{13}C (JMOD) NMR and IR spectra of compound **7a**

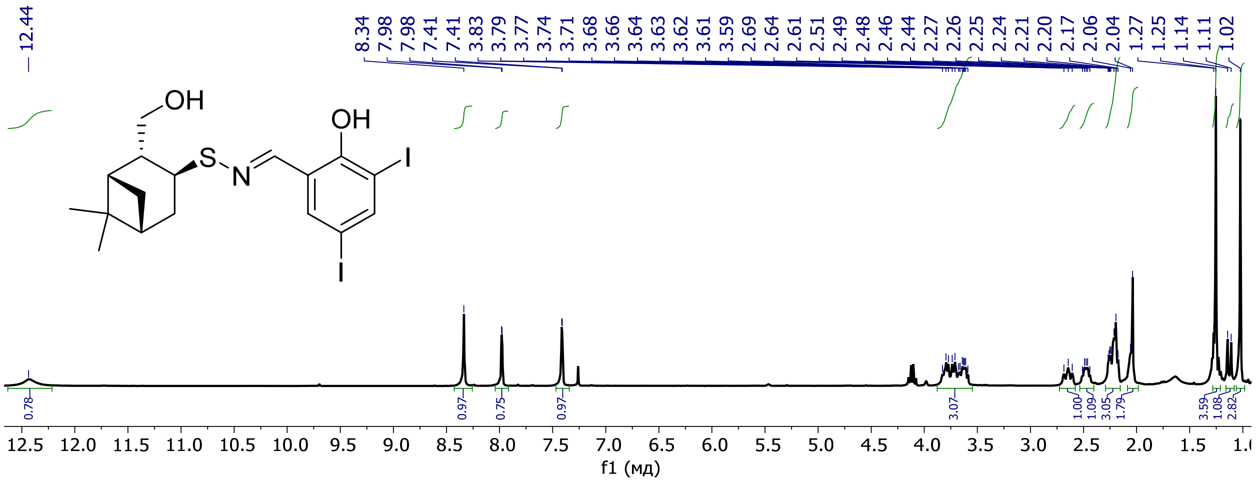

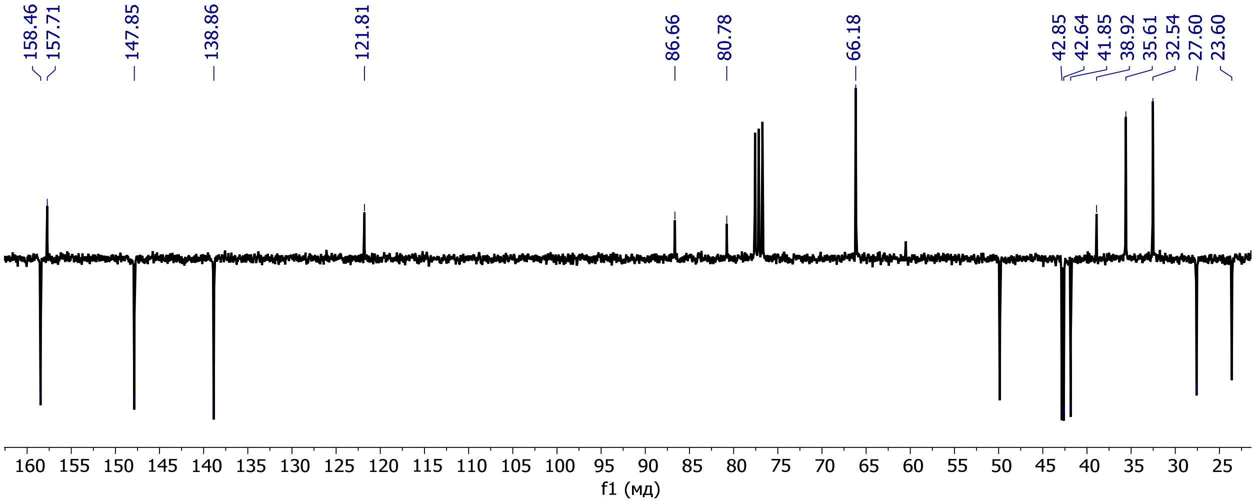

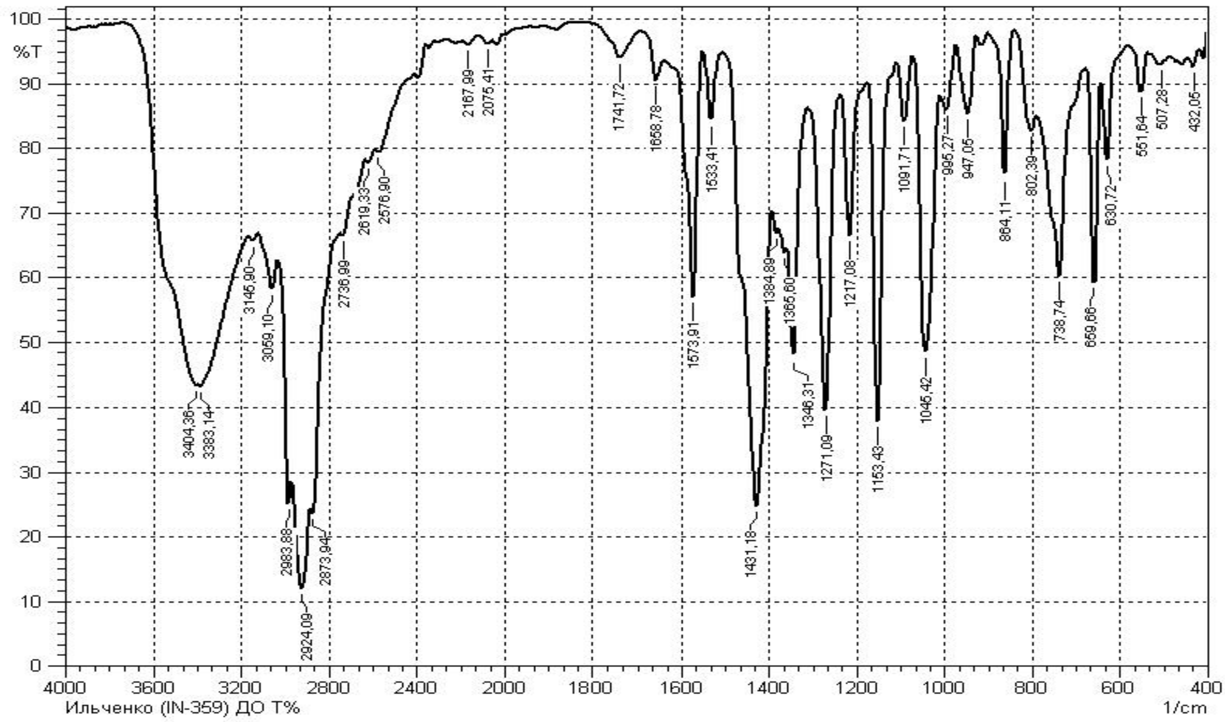

^{1}H, ^{13}C (JMOD) NMR and IR spectra of compound **8a**

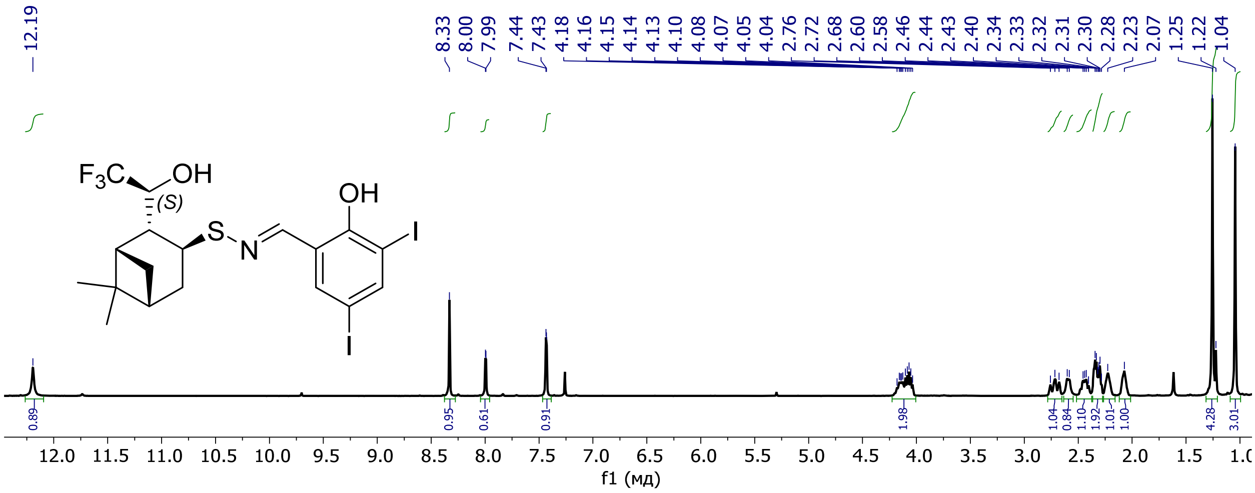

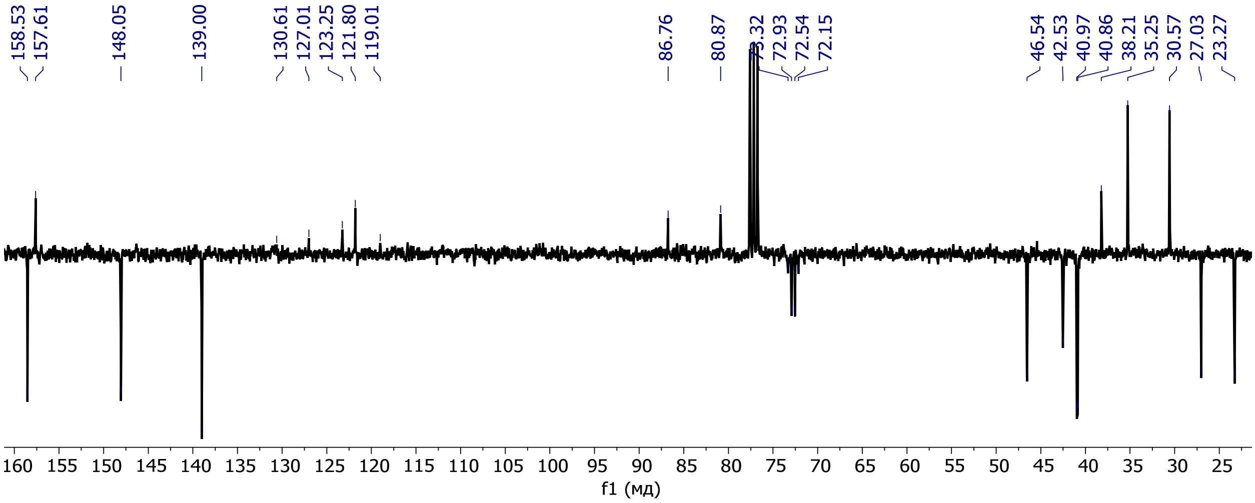

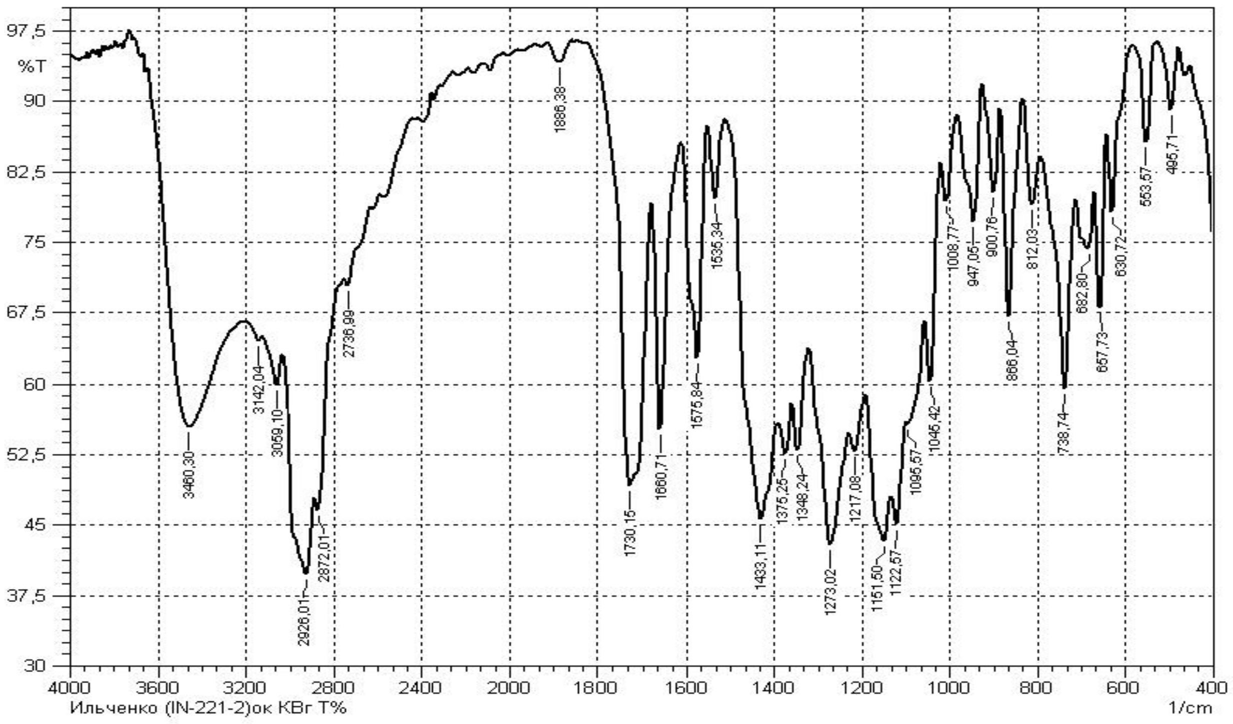

^{1}H, ^{13}C (JMOD) NMR and IR spectra of compound **9a**

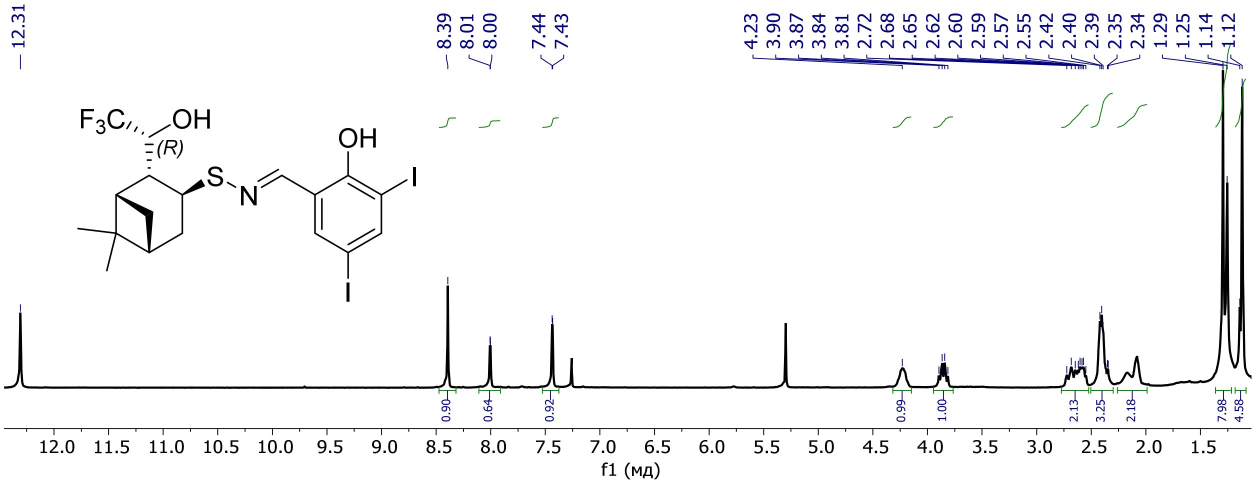

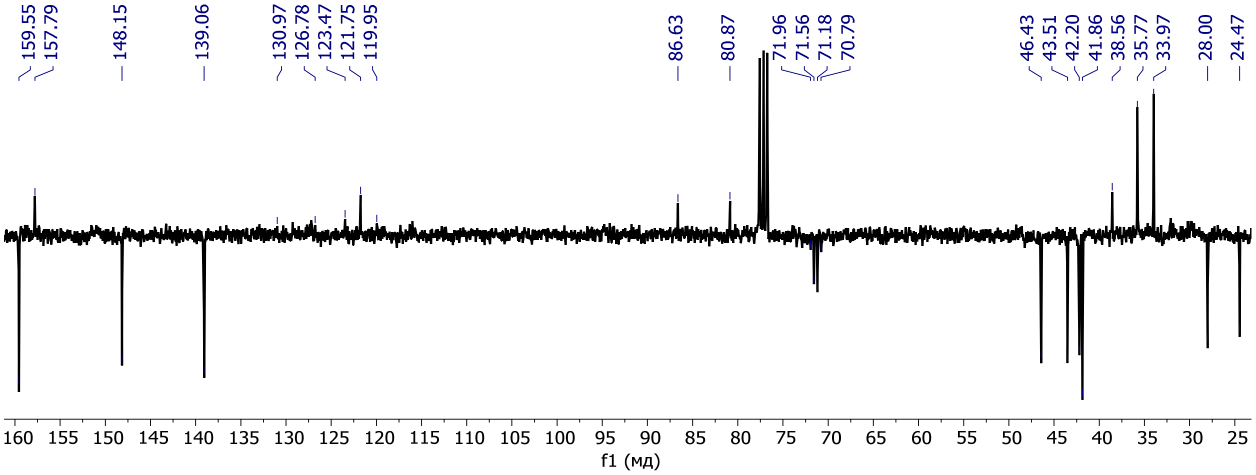

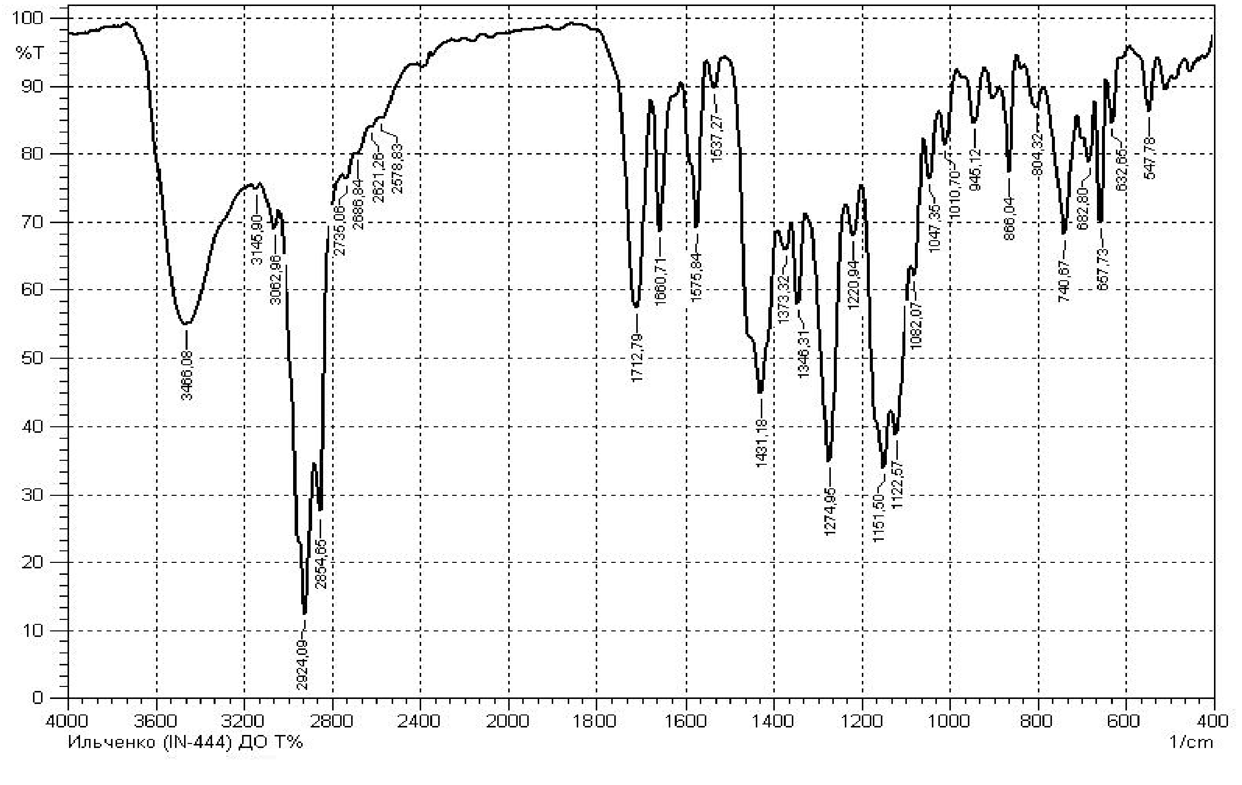

^{1}H, ^{13}C (JMOD) NMR and IR spectra of compound **7b**

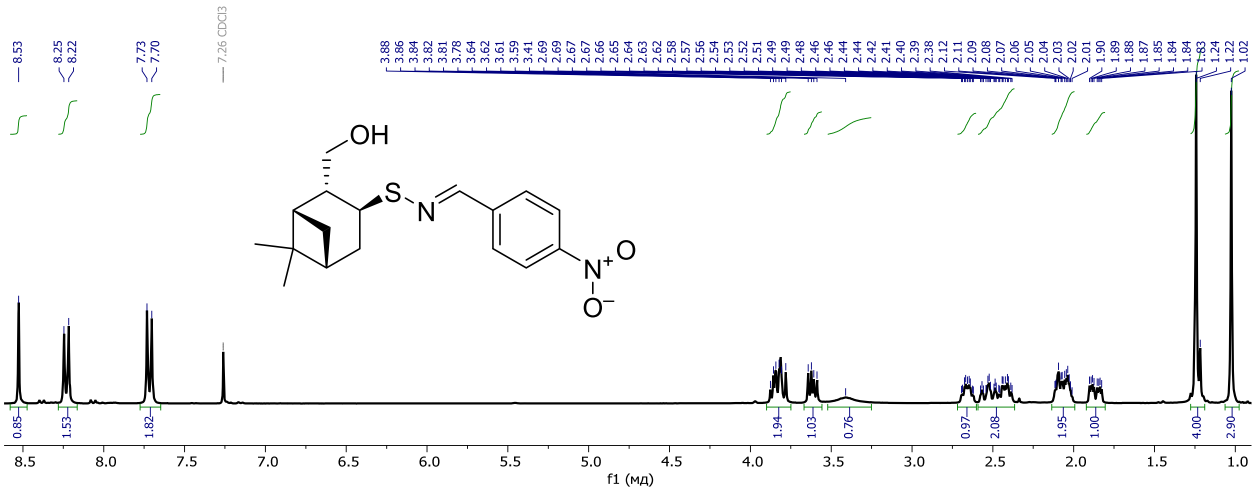

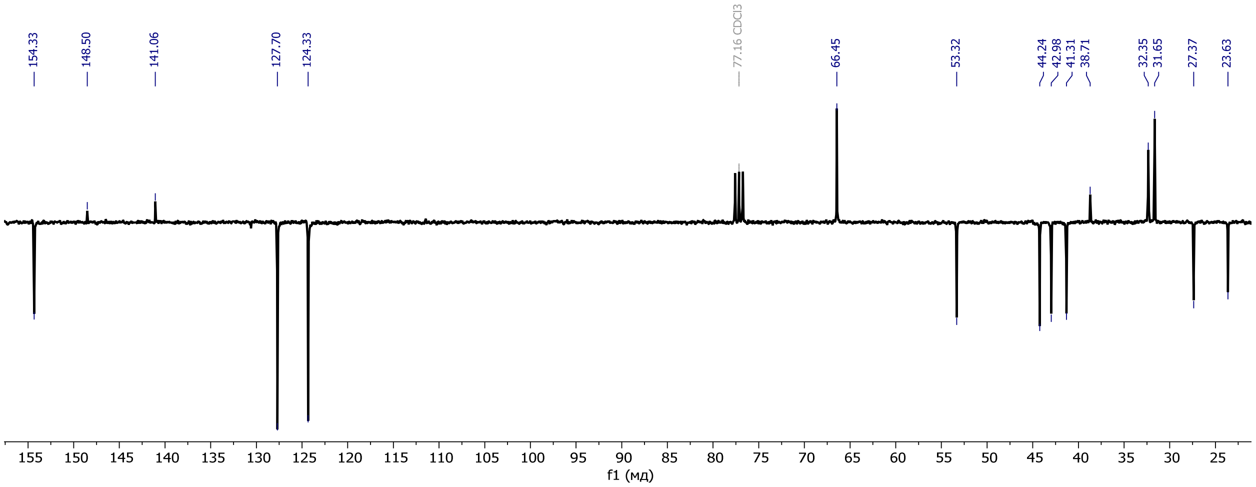

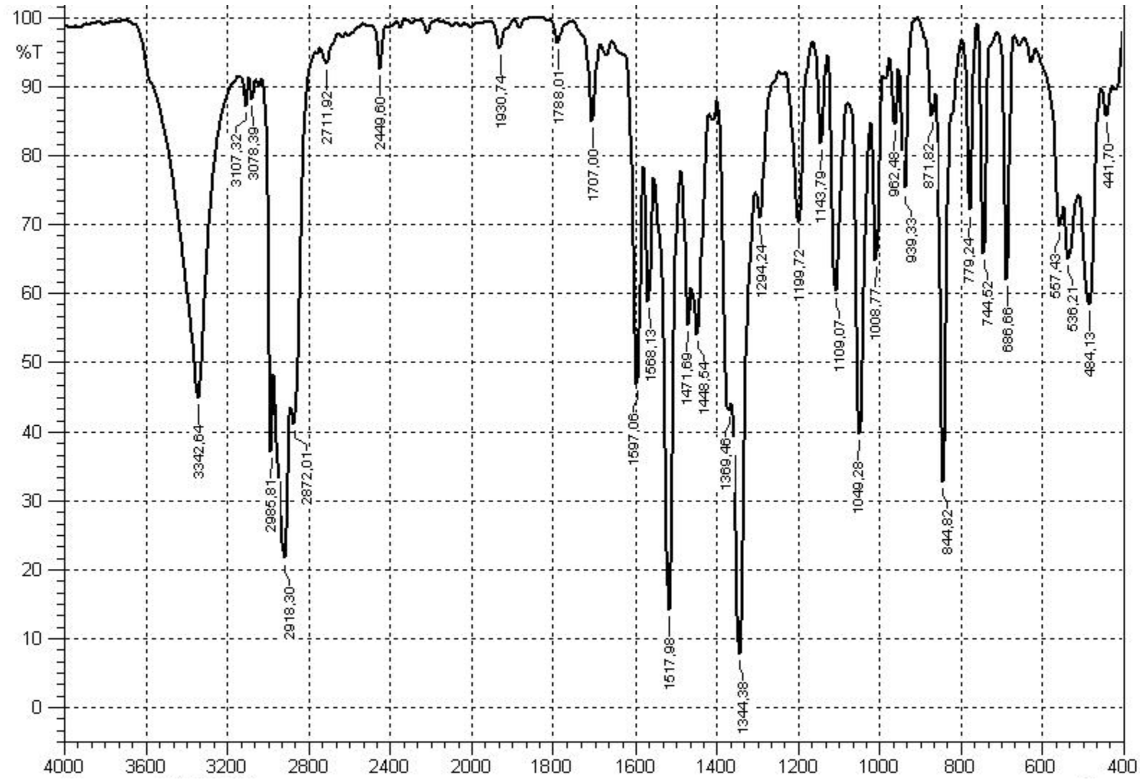

^{1}H, ^{13}C (JMOD) NMR and IR spectra of compound **8b**

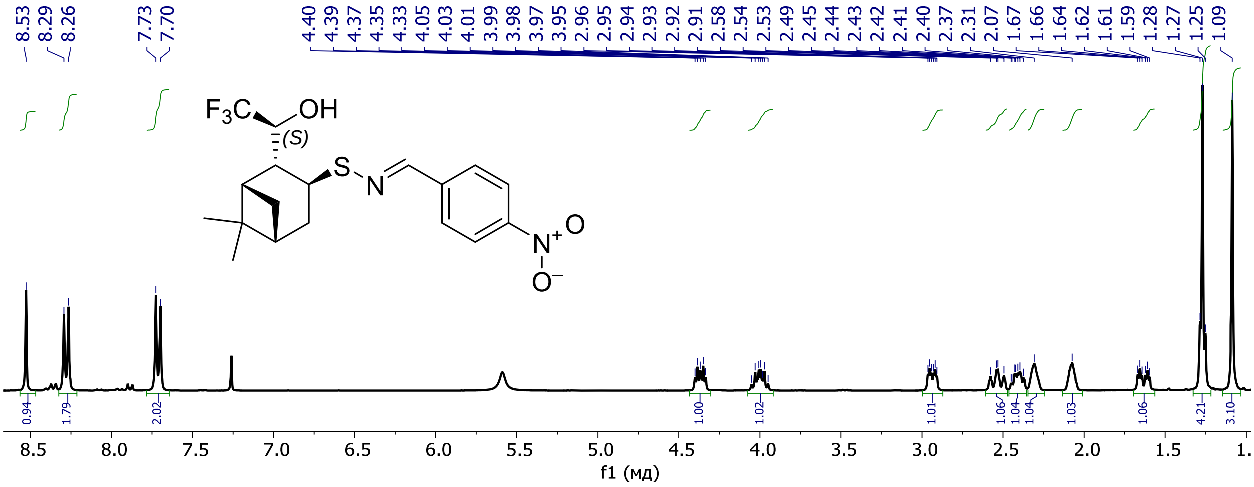

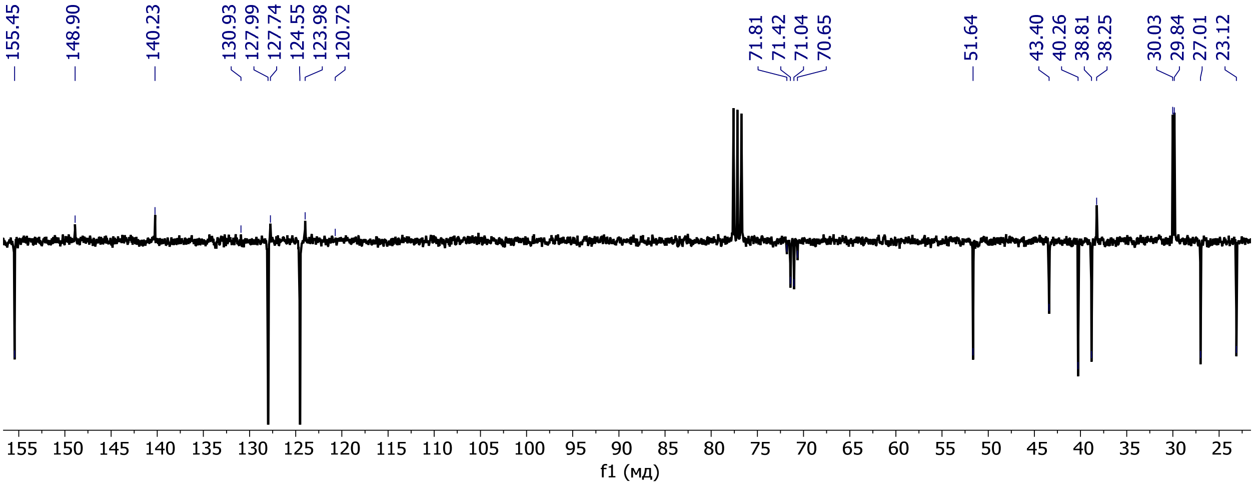

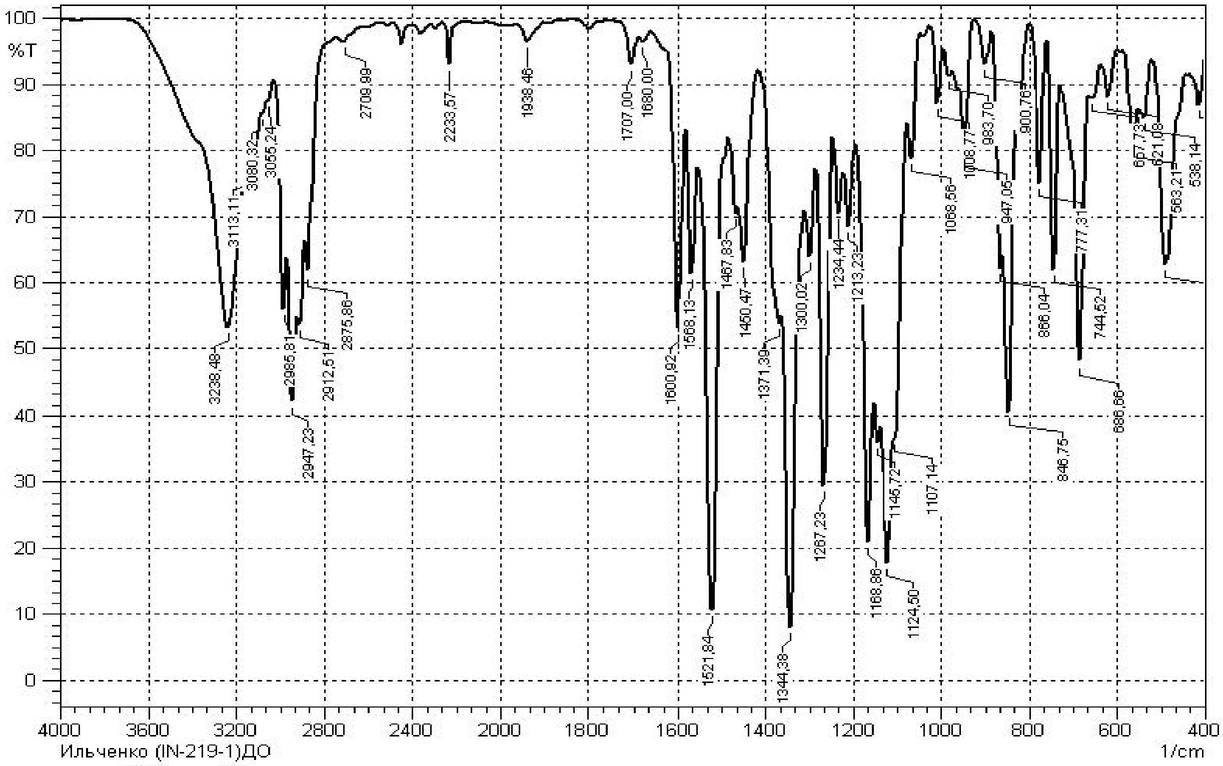

^{1}H, ^{13}C (JMOD) NMR and IR spectra of compound **7c**

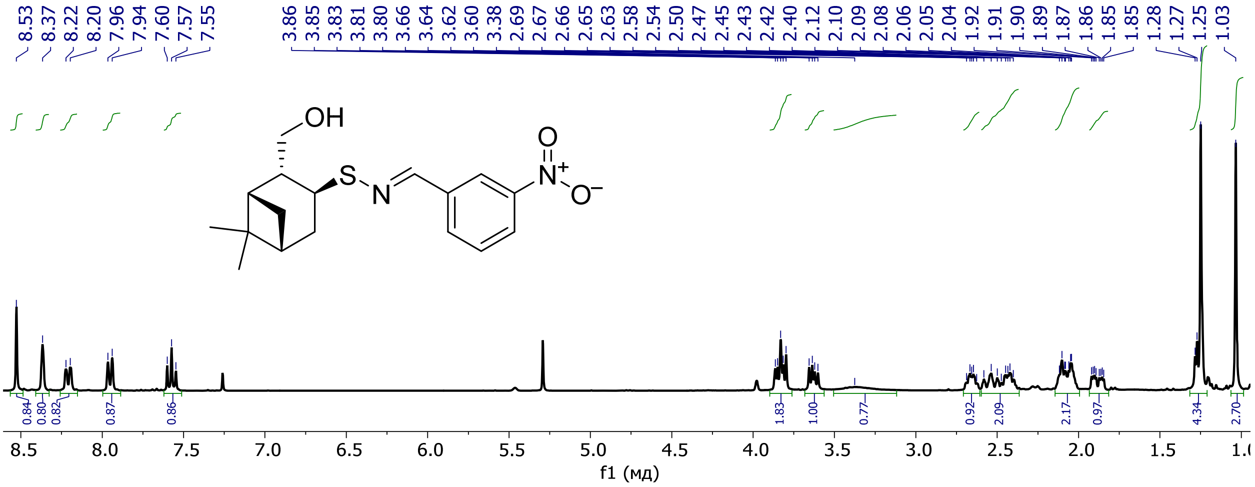

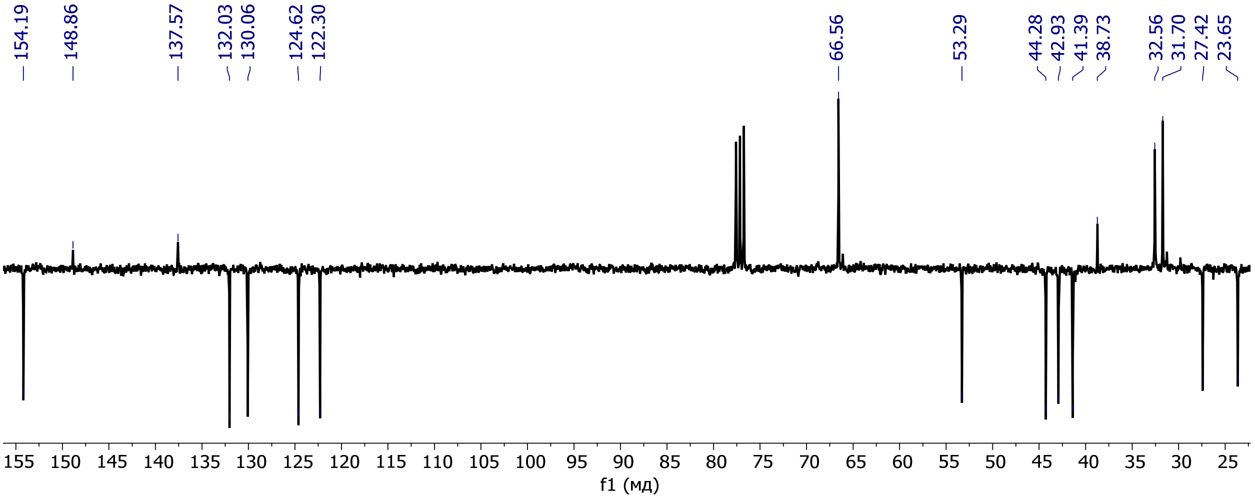

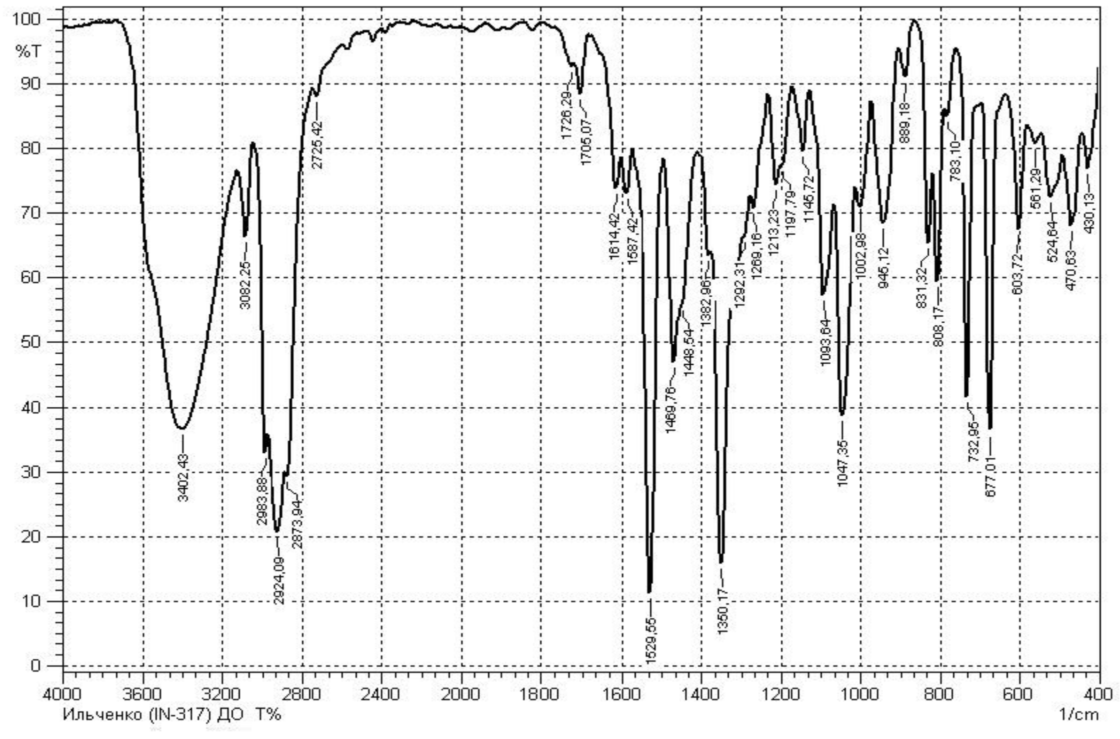

^1H, ^{13}C (JMOD) NMR and IR spectra of compound **8c**

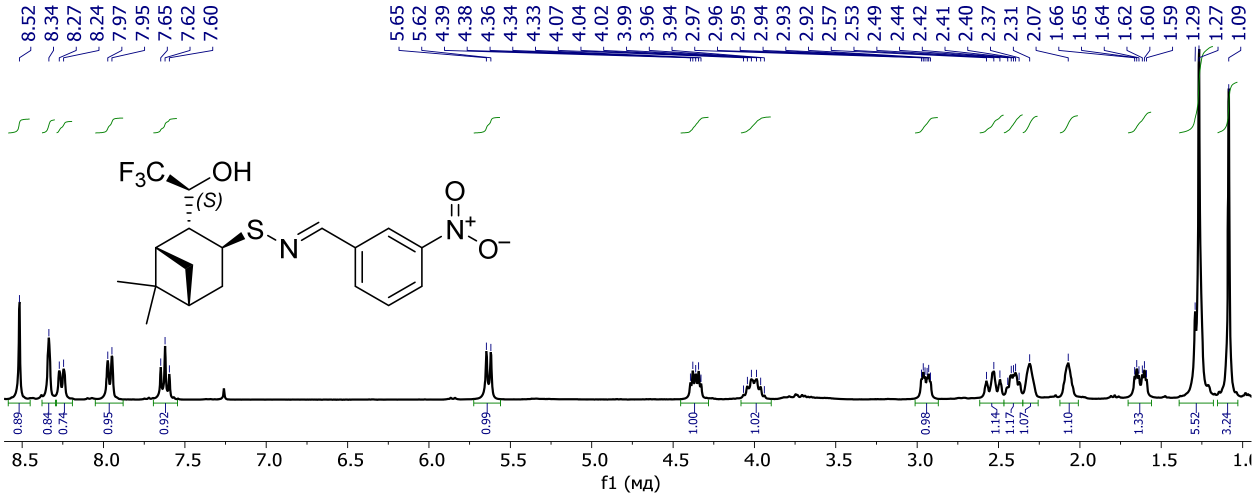

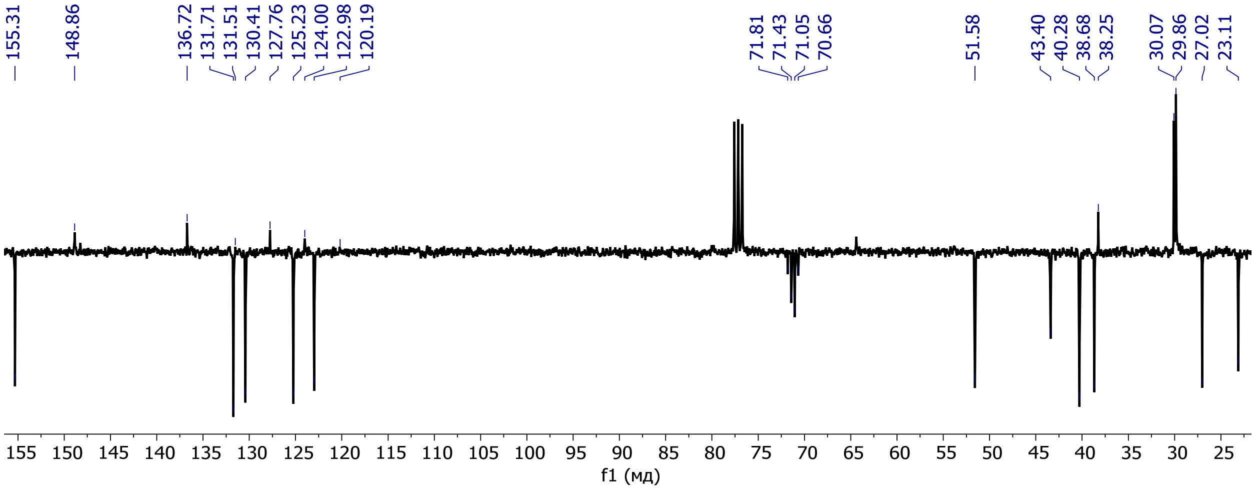

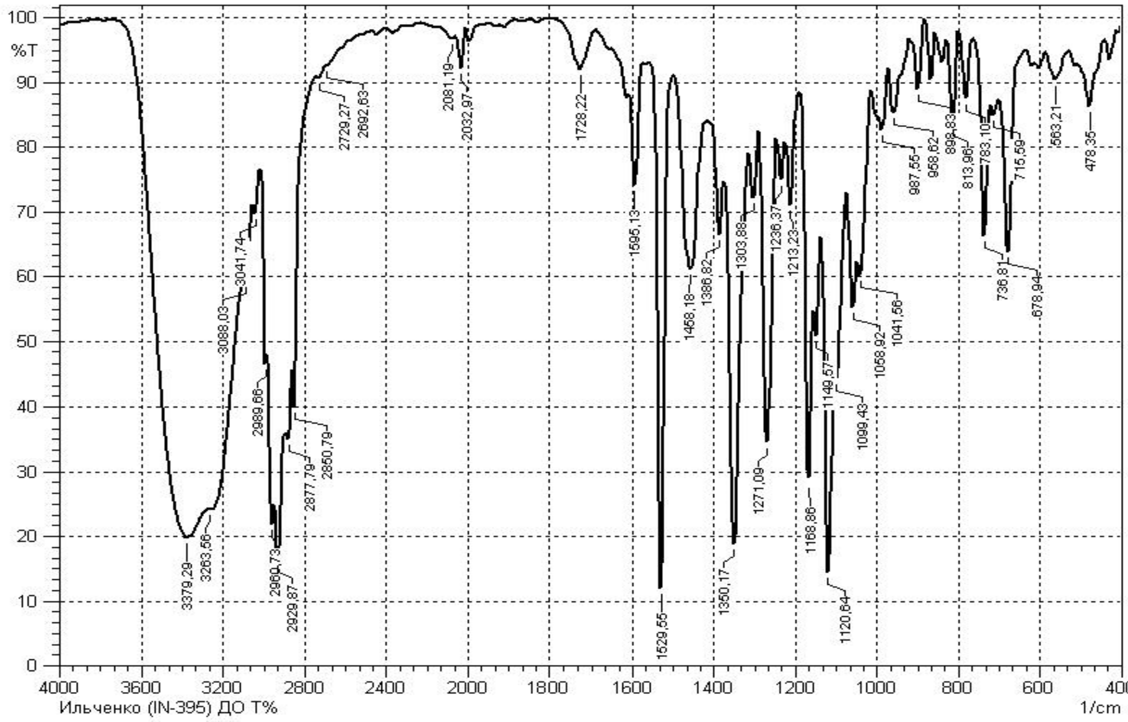

^{1}H, ^{13}C (JMOD) NMR and IR spectra of compound **7d**

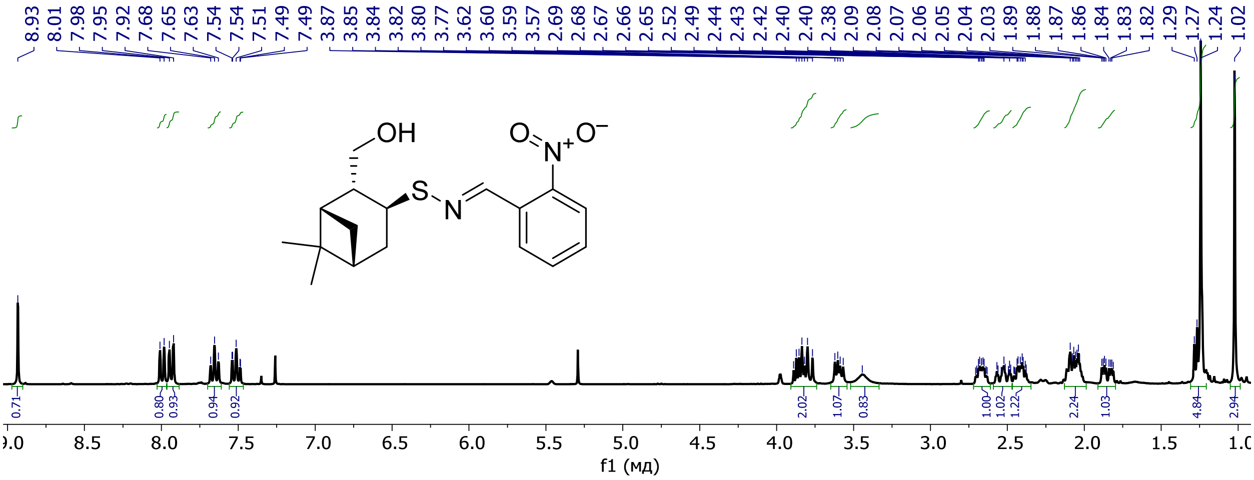

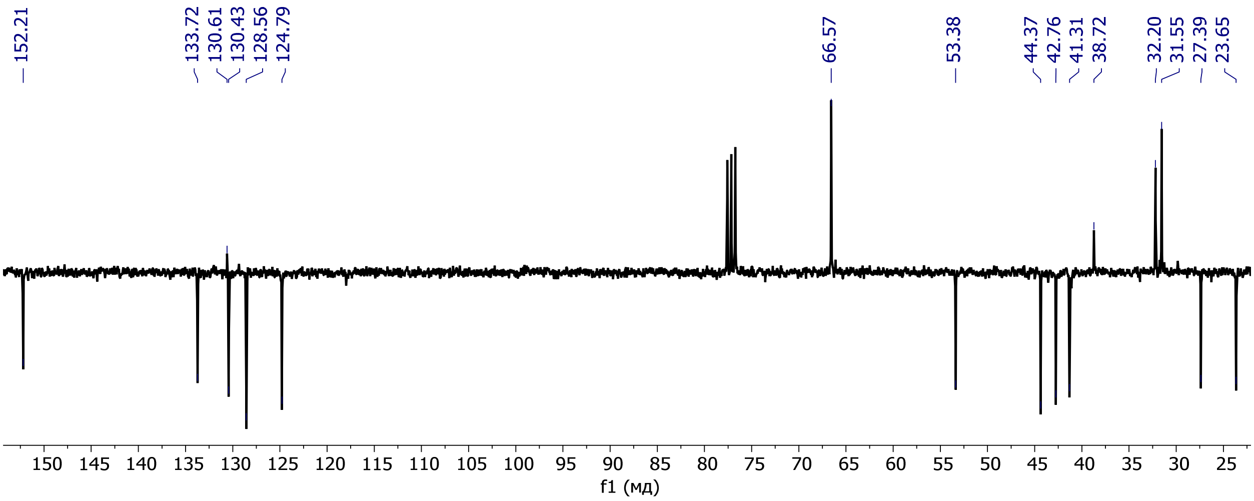

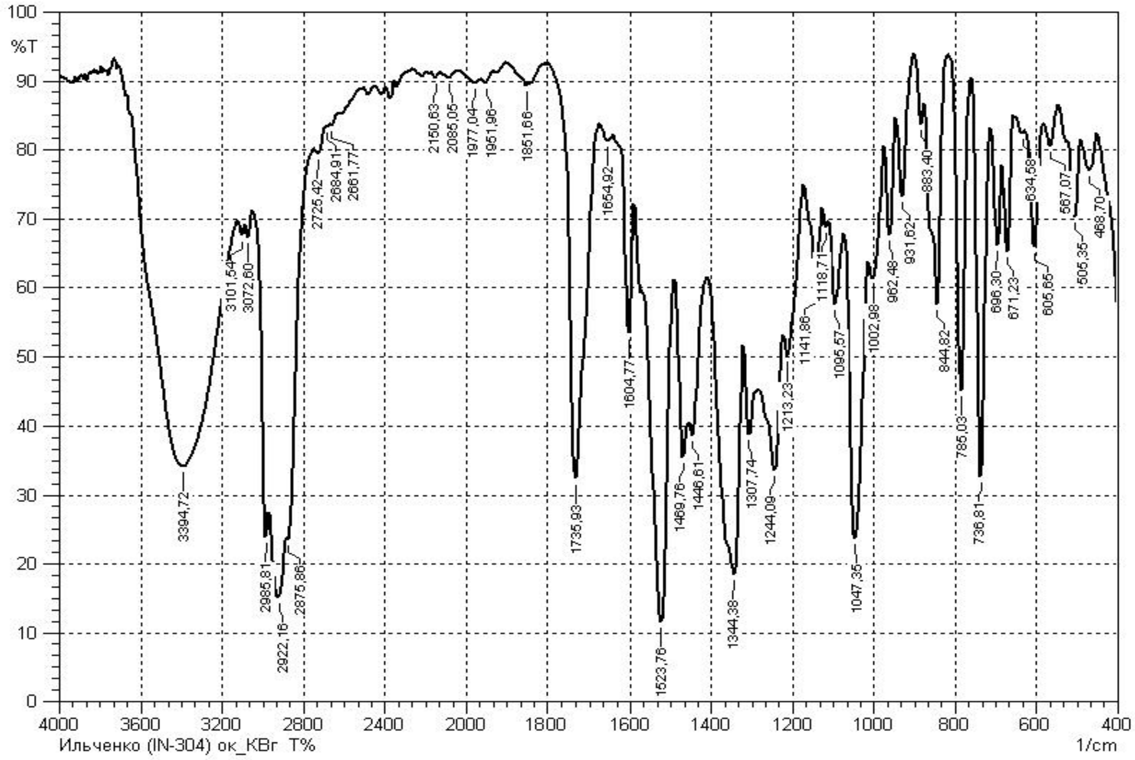

^{1}H, ^{13}C (JMOD) NMR and IR spectra of compound **8d**

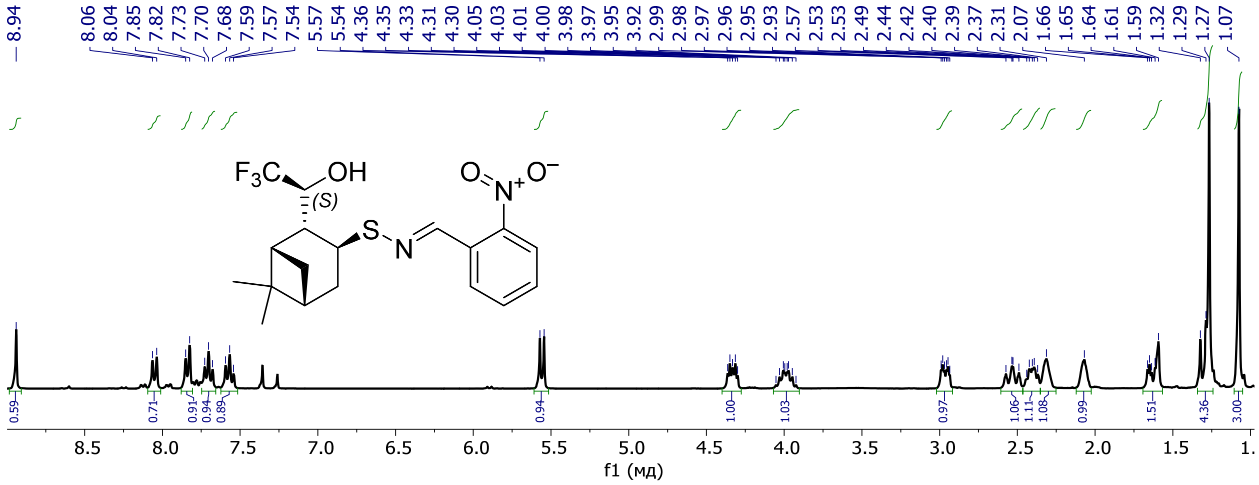

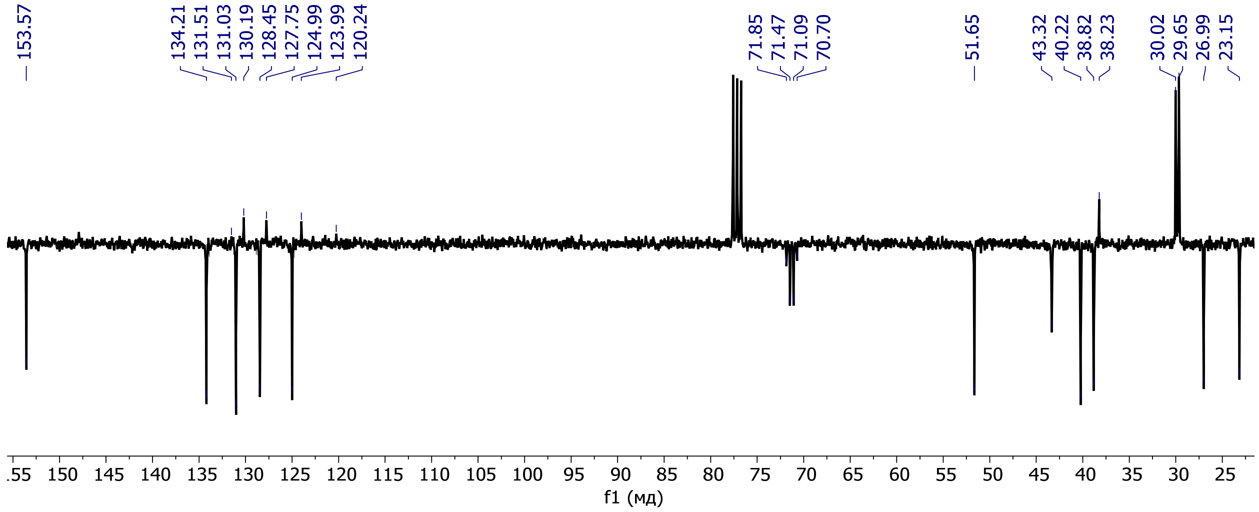

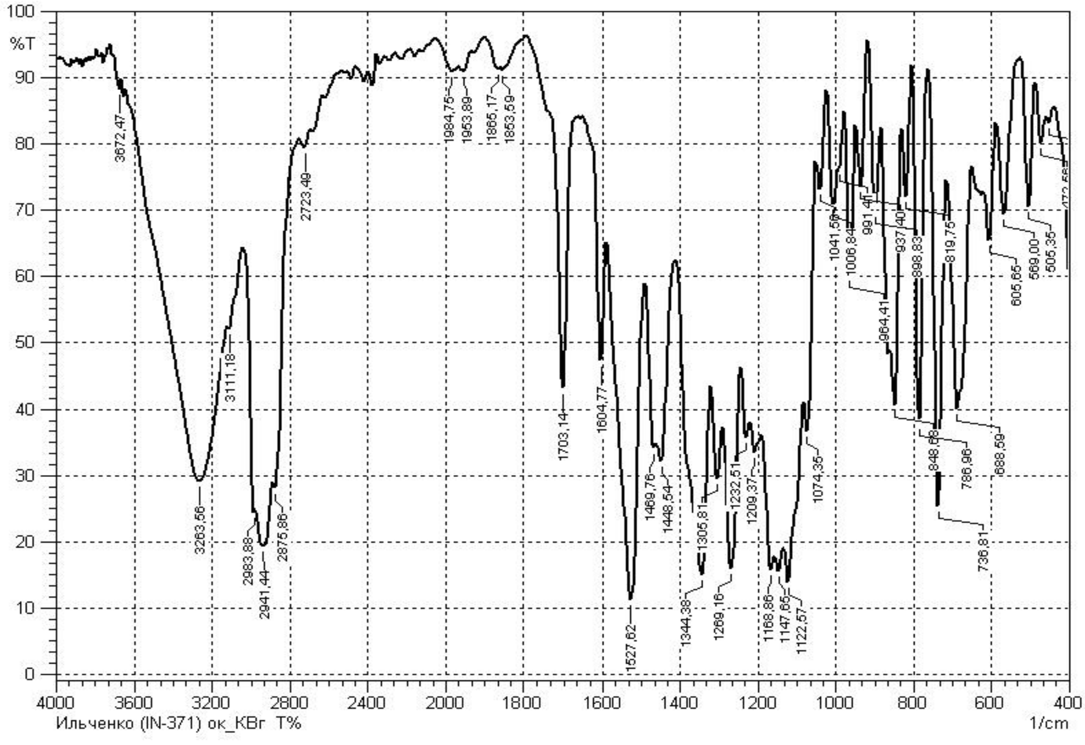

^{1}H, ^{13}C (JMOD) NMR and IR spectra of compound **7e**

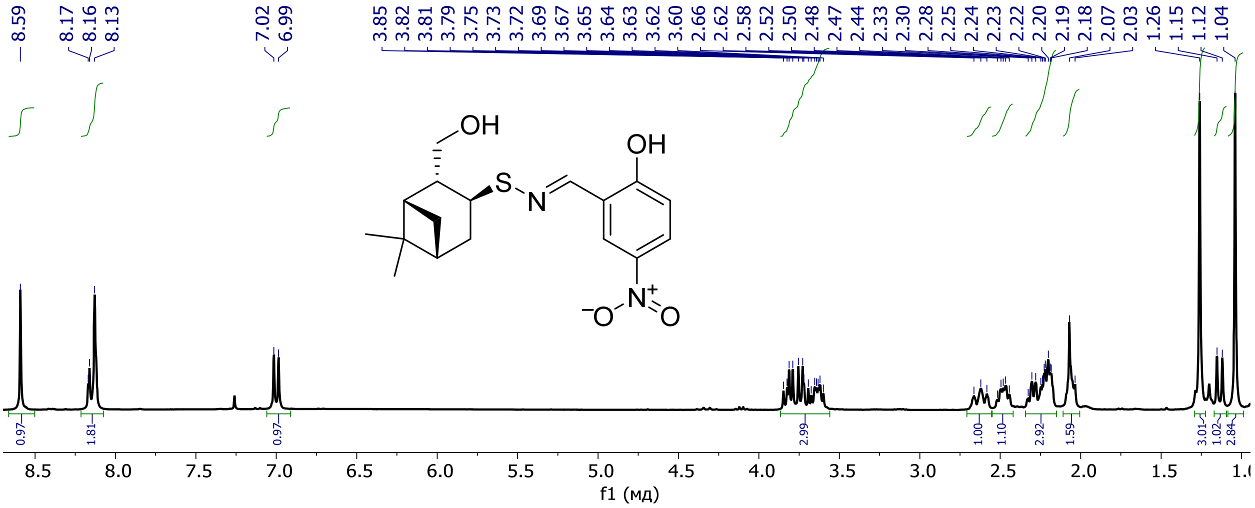

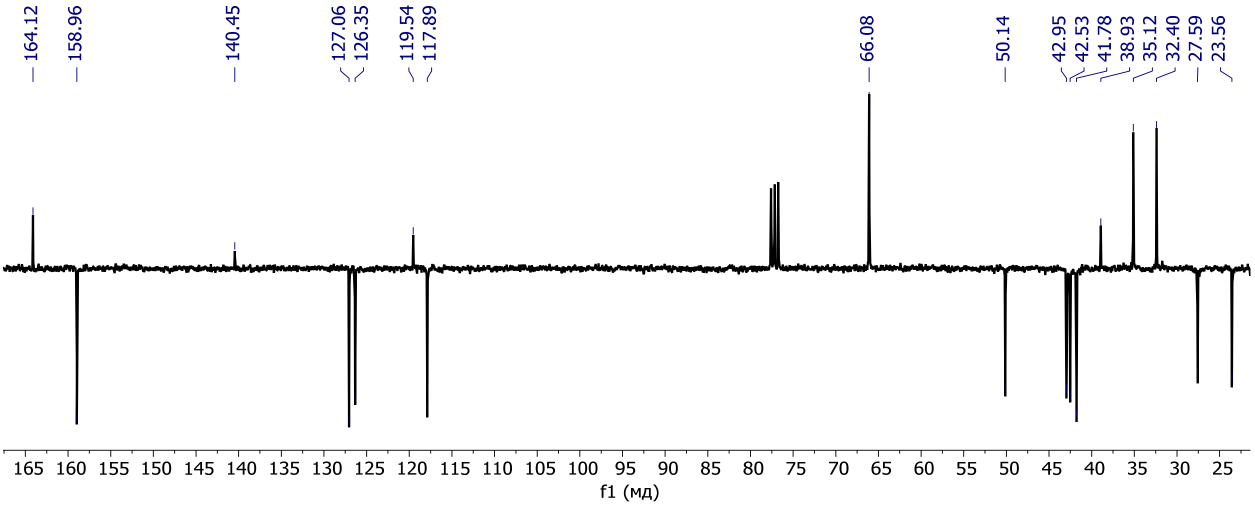

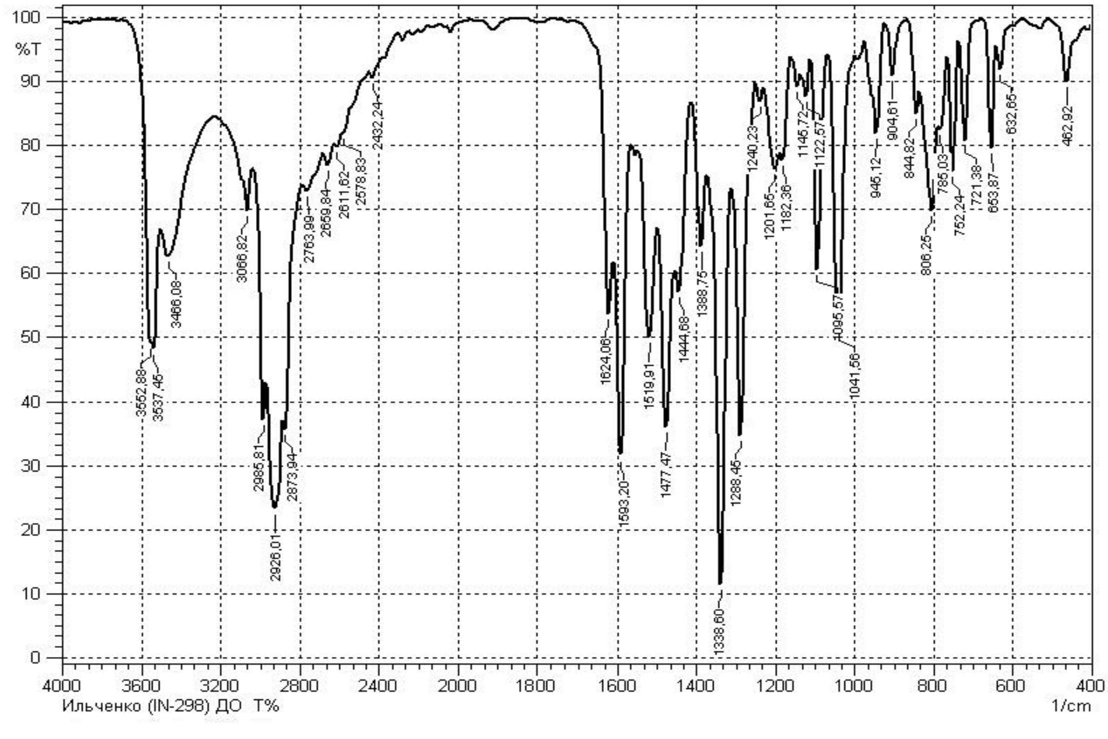

^{1}H, ^{13}C (JMOD) NMR and IR spectra of compound **8e**

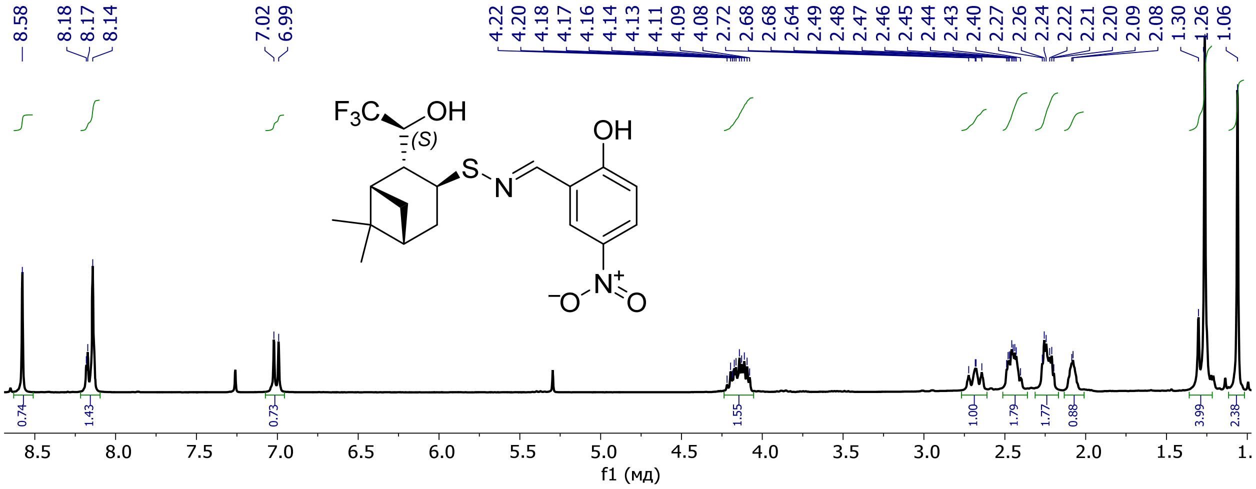

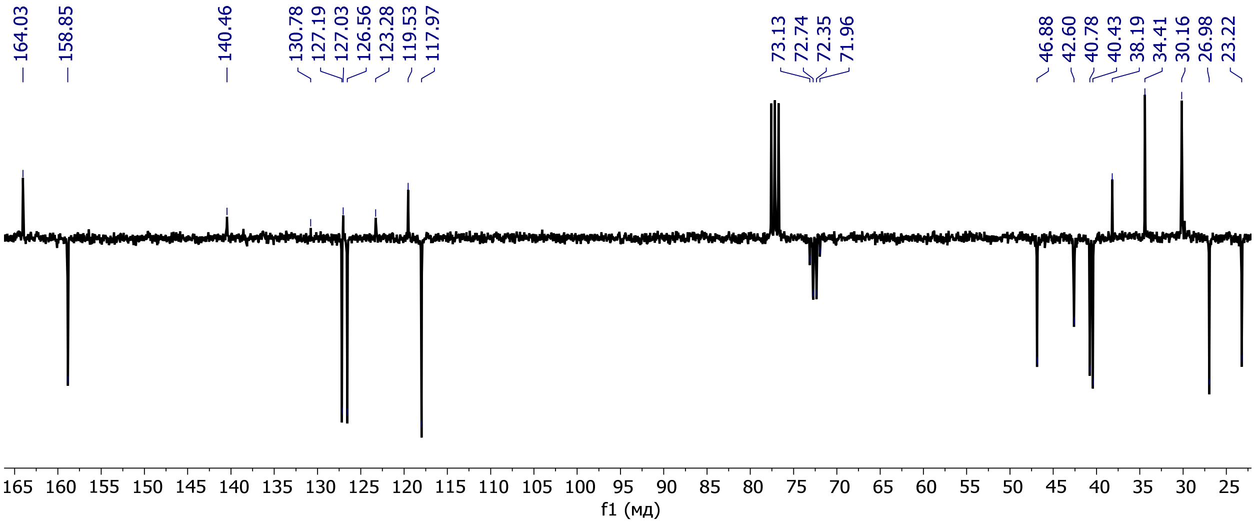

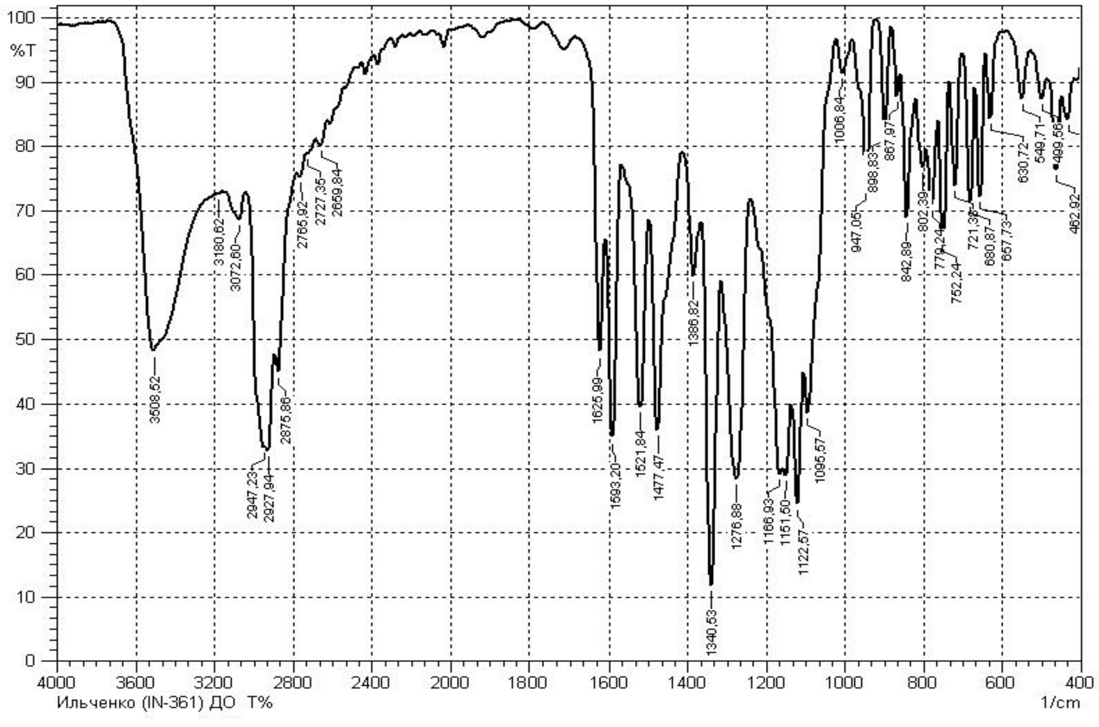

^{1}H, ^{13}C (JMOD) NMR and IR spectra of compound **7f**

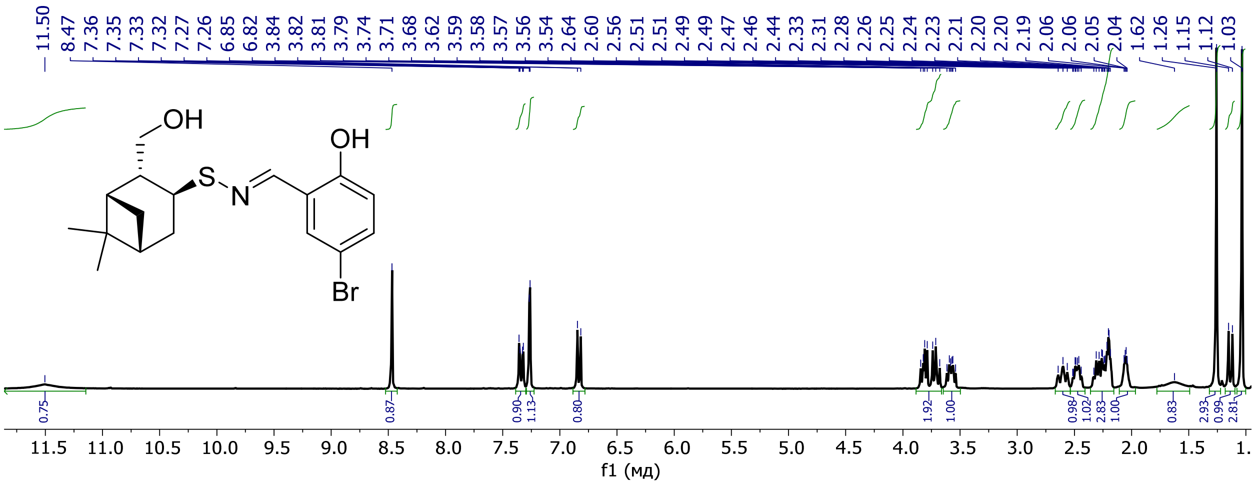

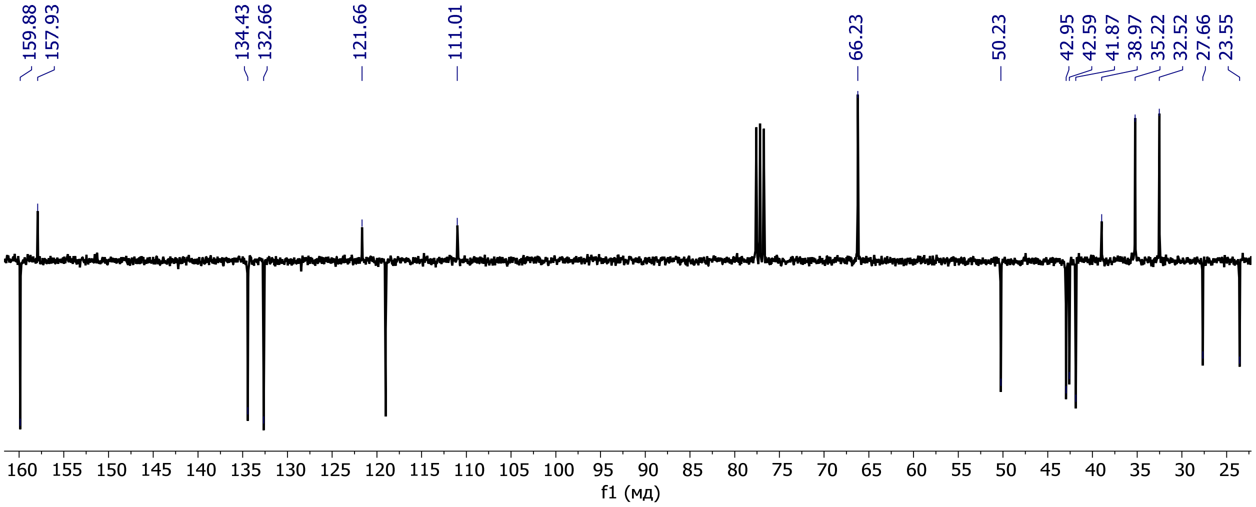

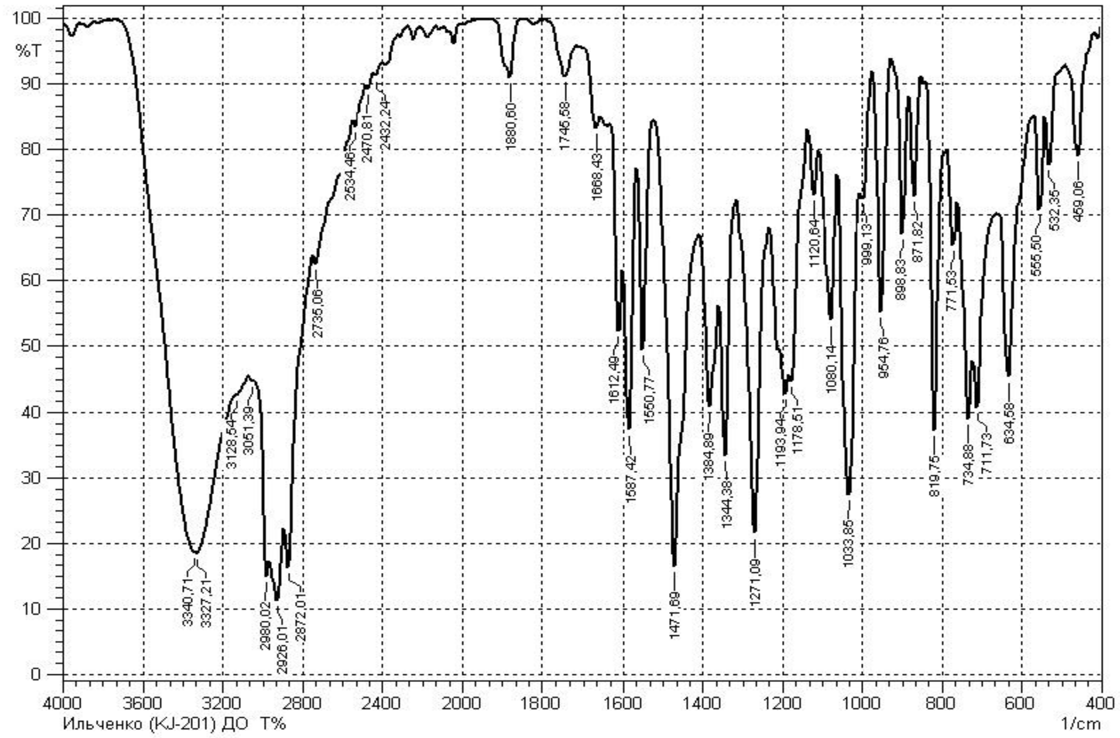

^{1}H, ^{13}C (JMOD) NMR and IR spectra of compound **8f**

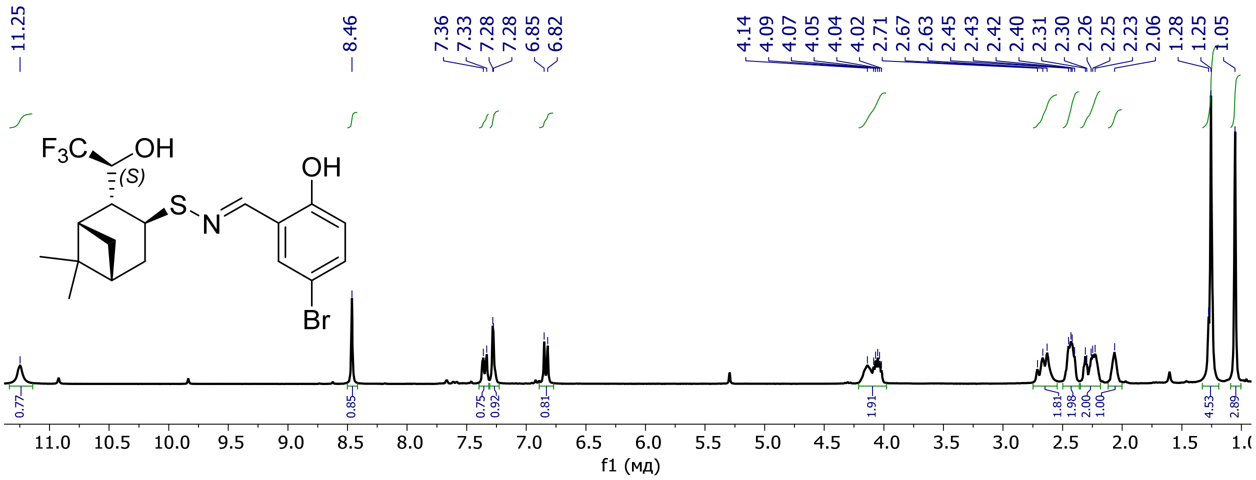

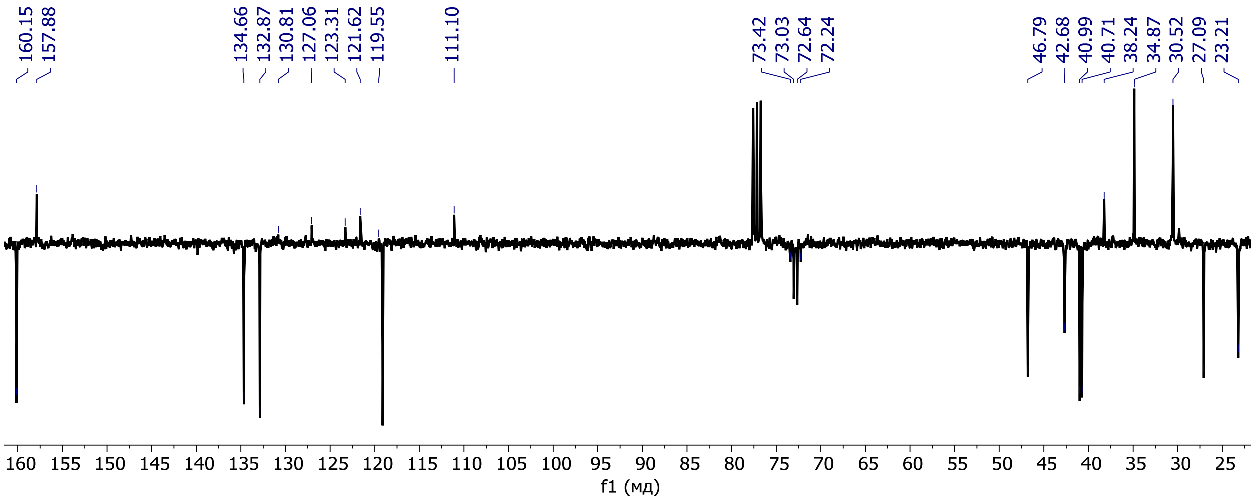

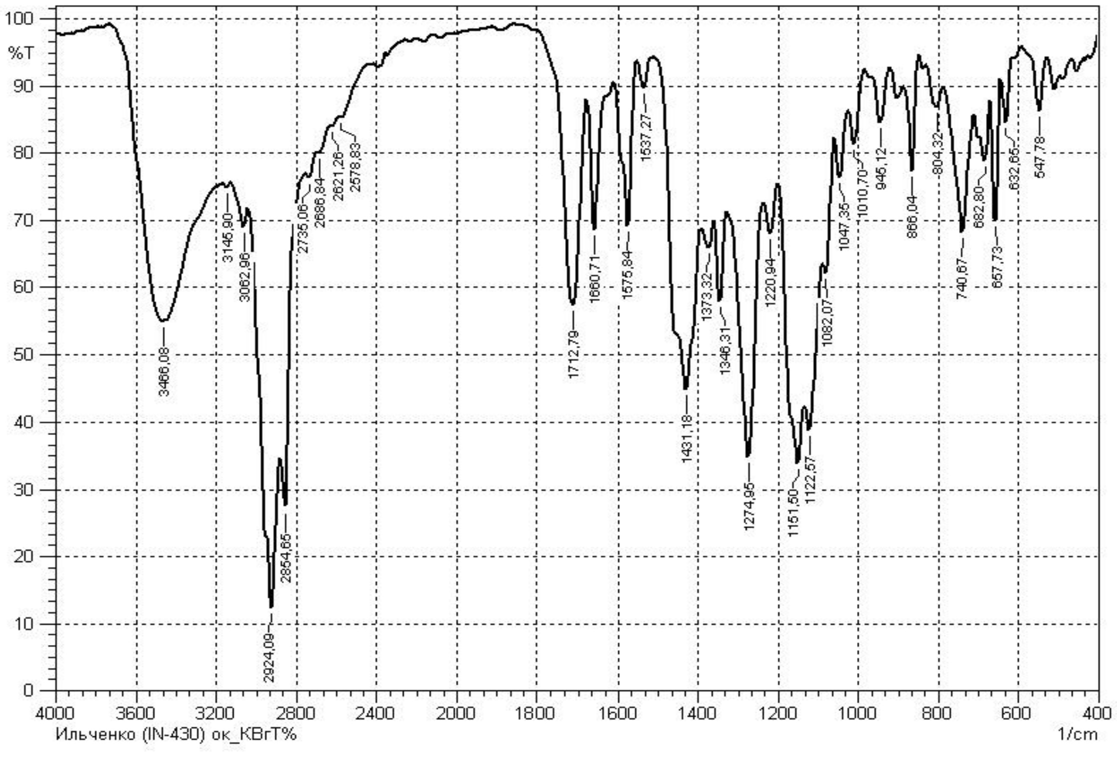

Supplement: Supplementary file 1 [file antibiotics-11-01548-s001.zip › antibiotics-1971312-supplementary.pdf]
